# From inserts to 3D spheroids: MAC-T and BME-UV1 co-culture models for in vitro reconstruction of the bovine mammary epithelial architecture

**DOI:** 10.1186/s13567-026-01763-5

**Published:** 2026-07-03

**Authors:** Julie Bellemans, Evelyne Meyer, Marion Boutinaud, Laurence Finot

**Affiliations:** 1 Laboratory of Biochemistry, Department of Veterinary and Biosciences, Faculty of Veterinary Medicine, Ghent University, Merelbeke, Belgium; 2 Laboratory of Applied Biotechnology, Department of Biotechnology, Faculty of Bioscience Engineering, Ghent University, Ghent, Belgium; 3 PEGASE, INRAE, Institut Agro Rennes-Angers, 35590 Saint-Gilles, France

**Keywords:** Mammosphere, mammary epithelial cells, spheroid, epithelial barrier, Transwell® insert, animal-friendly culture

## Abstract

Two bovine mammary epithelial cell lines, MAC-T and BME-UV1, were co-cultured either in Transwell® inserts or in 3D culture systems to comparatively investigate their potential to mimic the structure and function of a mammary alveolus. Using either Matrigel® or animal-friendly alternatives, i.e. rat collagen type I, a mixture of rat collagen type I and mouse laminin, Vitrogel® or matrix-free ultra-low attachment surfaces, both co-culture mammosphere development and tight junction formation were assessed. On Transwell® inserts, co-culture failed to establish a functional epithelial barrier. In contrast, varying patterns of cell–cell interaction and barrier organization emerged using the different 3D co-culture systems, providing new insights into the reconstruction of physiologically relevant and animal-friendly models of the bovine mammary gland.

**Supplementary Information:**

The online version contains supplementary material available at 10.1186/s13567-026-01763-5.

## Introduction, methods and results

Understanding the functioning of the bovine mammary gland is crucial, not only to elucidate mammary development and lactation, but also to better address mastitis, and to develop antimicrobial strategies. Mastitis is one of the most important diseases affecting dairy cattle, with major consequences for both animal welfare and economic performance. Having a functional in vitro model of the mammary alveolus would enable studying damages caused by mastitis and strategies to combat it.

**Figure 1:**
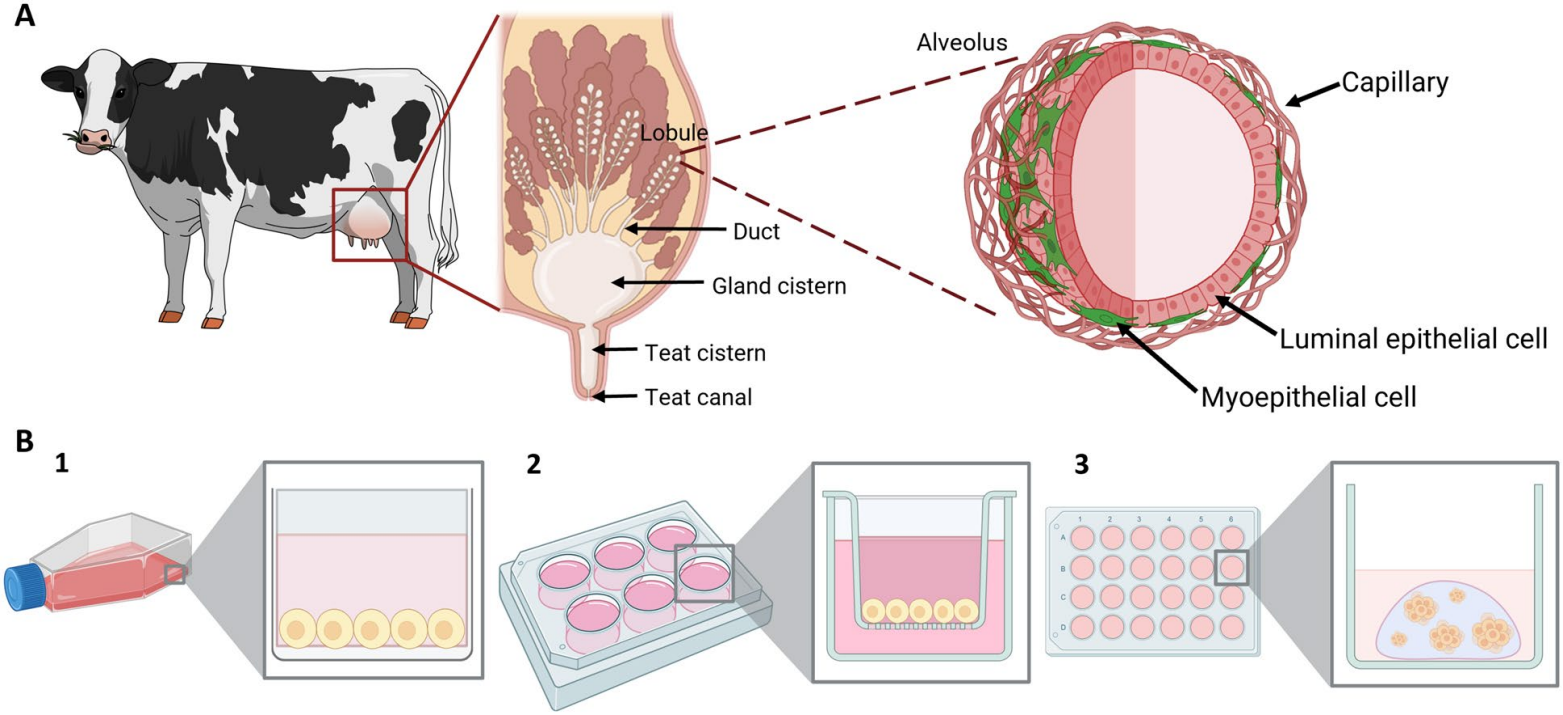
**The bovine mammary gland: architecture and in vitro experimental models. A ** Schematic representation of the bovine mammary gland anatomy from the udder (left) to the alveolus (right). Key structures include glandular tissue (alveoli, lobules, ducts), gland and teat cisterns, and teat canal. Diagrammatic cross-section of an alveolus illustrating the organization of mammary luminal and myoepithelial cells. **B** Overview of current in vitro cellular models of bovine mammary epithelial cells. Primary cells or immortalized cell lines (e.g., MAC-T and BME-UV1) can be cultured in 2D on coated plastic allowing cell adherence (**1**), on Transwell® membrane insert separating upper and lower compartments (**2**) or embedded in extracellular matrix to form 3D mammospheres (**3**) (created with Mind The Graph and BioRender).

The bovine mammary gland is a highly specialized organ responsible for the synthesis and secretion of milk. The milk-producing tissue is composed of alveoli, considered as the gland’s functional milk-producing units, organized into lobes and lobules. Each alveolus is constituted by a layer of polarized mammary epithelial cells (MECs), its apical surface facing the milk-containing lumen, whereas the basal surface stands in contact with contractile myoepithelial cells and the basal lamina. This plurilayered epithelium is embedded in a complex stromal environment consisting of extracellular matrix (ECM) proteins, fibroblasts, adipocytes, lymphatics, and blood vessels (Figure 1A) [1].

Although in vivo studies remain a reference standard for studying mammary physiology, the use of animals in research is costly, raises major ethical concerns and is therefore increasingly subjected to strict regulations, often resulting in low replication numbers compromising scientific accuracy [2]. Consequently, in vitro models are widely used as complementary tools to explore mammary gland function under controlled and reproducible conditions. However, reproducing the milk-producing capacity and gland-like architecture of secretory alveoli in vitro remains a major challenge in mammary biology. In order to facilitate such studies, immortalized bovine MECs (bMECs) lines have been established. The most commonly used bMECs are the MAC-T (Mammary Alveolar Cells-Transformed) and BME-UV1 (Bovine Mammary Epithelial—University of Vermont 1 [3]) cell lines, which present distinct epithelial cell subtypes. MAC-T cells exhibit a basal phenotype characterized by high expression of the cell surface marker CD49f and keratin (K) 14, which is known to be expressed by basal cells [4]. In contrast, BME-UV1 cells display a luminal phenotype, expressing K19, K7 or K8, EpCAM and aldehyde dehydrogenase (ALDH) [3–6]. In addition to these immortalized cell lines, primary bMECs can also be used; however, with limiting factors being their replicative senescence, restrictive long-term proliferation and poor experimental reproducibility [7].

Most commonly, bMECs are cultured in two-dimensional (2D) systems on flat plastic surfaces (Figure 1B.1). However, in such conditions, the cells cannot establish essential interactions with the ECM, which is lacking and fail to acquire polarity, and therefore their functionality. Culturing bMECs in more advanced systems such as three-dimensional (3D) environments including Transwell® inserts, can help preserve or restore critical cell–cell and cell–matrix interactions, supporting the development of more complex tissue-like architecture, appropriate signaling, polarity, and barrier function [2, 8, 9].

Few studies reported the use of Transwell® insert for bMECs. In Transwell® models, cells are grown on a permeable membrane separating an upper and a lower compartment, mimicking the cell polarization of the mammary epithelium. In the mammary gland, tight junctions regulate paracellular transport and maintain the integrity of the blood-milk barrier [10, 11]. To reconstitute this in vivo situation, Tsugami et al. (2020) cultured primary bMECs on permeable membrane inserts (Figure 1B.2) [8, 12]. In this culture model, primary bMECs were shown to form tight junctions and displayed features of a polarized and functionally differentiated epithelium, demonstrating their suitability for studying mammary barrier function under lactation-mimicking conditions.

Several other 3D culture systems have been developed in order to better reproduce the in vivo architecture and differentiation of bovine mammary epithelium. Embedding bMECs in extracellular matrices such as Matrigel® promotes the formation of spheroids (subsequently named mammospheres) or acini-like structures that more closely resemble alveolar organization and foster polarization and lumen formation, offering a more physiological context than conventional 2D models [4, 13–15]. In 2009, Kozlowski et al. demonstrated the ability of BME-UV1 cells to form polarized mammospheres after 16 d of culture within Matrigel® (Figure 1B.3). These structures exhibited epithelial polarity, confirmed by Zonula occludens-1 (ZO-1) and E-cadherin localization and transcriptomic profiles consistent with epithelial function and polarization [13]. Since then, three studies have reported that 3D culture with Matrigel® promotes a more differentiated and functional phenotype of bMECs compared to standard 2D culture. One study reported that primary bMECs structured into mammosphere expressed milk synthesis genes [14], while Zhan et al. (2017) established immortalized bMECs from lactating cows that formed mammospheres with enhanced polarization (β-catenin/E-cadherin localization) [15]. Additionally, our group studied the behavior of BME-UV1 and MAC-T cell lines cultured in 3D systems, also based on Matrigel®, and compared them to matrix-free ultra-low attachment (ULA) plastic [4]. We observed that 3D culture revealed divergent identities and functional properties of these two bMEC lines: while BME-UV1 cells formed organized spherical mammospheres, consistent with a progenitor-like luminal phenotype, MAC-T cells displayed limited spatial organization, reflecting their basal/myoepithelial characteristics [4]. Despite the potential advantages of combining these luminal and basal cell types in culture, such a 3D co-culture model has not yet been reported.

The primary objective of this study was to compare the co-culture of luminal (BME-UV1) and basal (MAC-T) cells under different 3D culture conditions, including Transwell® and Matrigel® embedding models. Secondary objectives were (i) to evaluate the formation of tight junctions in these models (along with epithelial barrier readouts for culture using Transwell®) and (ii) to evaluate animal-friendly alternatives to Matrigel®, namely collagen type I (collagen), a 1:1 (v/v) mixture of collagen type I and mouse laminin (collagen + laminin), Vitrogel®, and matrix-free ULA plates.

### (Co-cultured) MAC-T and BME-UV1 cell lines fail to form a cell barrier and tight junctions on Transwell® insert

Before performing co-culture of bMECs on Transwell® inserts or in 3D culture systems, we first defined a proliferation and a differentiation medium suitable for the two bMECs lines. These preliminary co-culture experiments were first performed on flat plastic (2D culture). BME-UV1 and MAC-T cells were simultaneously co-cultured in either MAC-T medium, BME-UV1 medium or a mixture of BME-UV1 and MAC-T media in equal proportions (1:1 v/v) (2 days (d) in proliferation medium followed by 3 d in differentiation medium). Cell morphology and progression to confluence were observed by histology in a 2D monolayer. After trypsinization, cells were systematically stained with Trypan blue at a final concentration of 0.2% (Bio-Rad, Marnes-la-Coquette, France), counted to assess cell viability (% live/dead cells) using a TC20 Automated Cell Counter (Bio-Rad, Marnes-la-Coquette, France, see Additional file 2A for references and supplier information). The culture conditions and immunostaining protocols for MAC-T and BME-UV1 cells were based on the previous report of Arévalo Turrubiarte et al. (2016) (see composition of media in Additional file 2A and antibody specifications in Additional file 2B) [4].

When BME-UV1 and MAC-T cells were simultaneously co-cultured in either MAC-T or BME-UV1 medium, the MAC-T cells dominated in MAC-T medium, whereas BME-UV1 cells dominated in BME-UV1 medium, indicating a competitive growth advantage in their respective culture medium (Additional file 1A and B). In contrast, when BME-UV1 and MAC-T media were mixed in equal proportions (1:1 v/v), both cell types proliferated to a similar extent, with no visible signs of apoptosis (Additional file 1C). In the mixed medium, we observed cell proliferation (detected after Ki67 labeling shown in Additional file 1D), expected morphology, progression to confluence and low percentage of cell death (5–14%) (Additional file 1E and F). Therefore, this combined medium was used in all subsequent co-culture experiments.

To acquire reference values for bMEC lines grown on Transwell® inserts (polyester membrane inserts; 0.4 μm pore size; 1.1 cm^2^ surface area; Sarstedt France, Marnay, France), we used primary bMECs isolated from the mammary tissue of a 17-month-old Holstein heifer as a positive control for transepithelial electrical resistance (TEER) measurement, Lucifer yellow (LY) assay and ZO-1 labeling. Cryopreserved mammary tissue was enzymatically digested to single cells and sorted as previously described using an antibody anti-CD49f, which is known to be a cell surface marker of epithelial cell type (Additional file 2B and C) [16]. CD49f + cells were seeded on Transwell® inserts and cultured for 14 d. To evaluate the epithelial barrier integrity in vitro, both primary bMECs and bMEC lines were grown in their respective optimized proliferation medium (see composition of media in Additional file 2A) on inserts placed in 12-well plates at a density of 1 × 10^5^ cells and incubated at 37 °C under 5% CO_2_. The following day, the proliferation medium was replaced by a differentiation medium, renewed every 2 d (1.5 mL in the lower, 1 mL in the upper compartment). In addition, epithelial barrier formation was evaluated under the same experimental conditions using collagen-coated and Matrigel-coated Transwell® inserts (see Additional file 2A for coating procedure).

**Figure 2:**
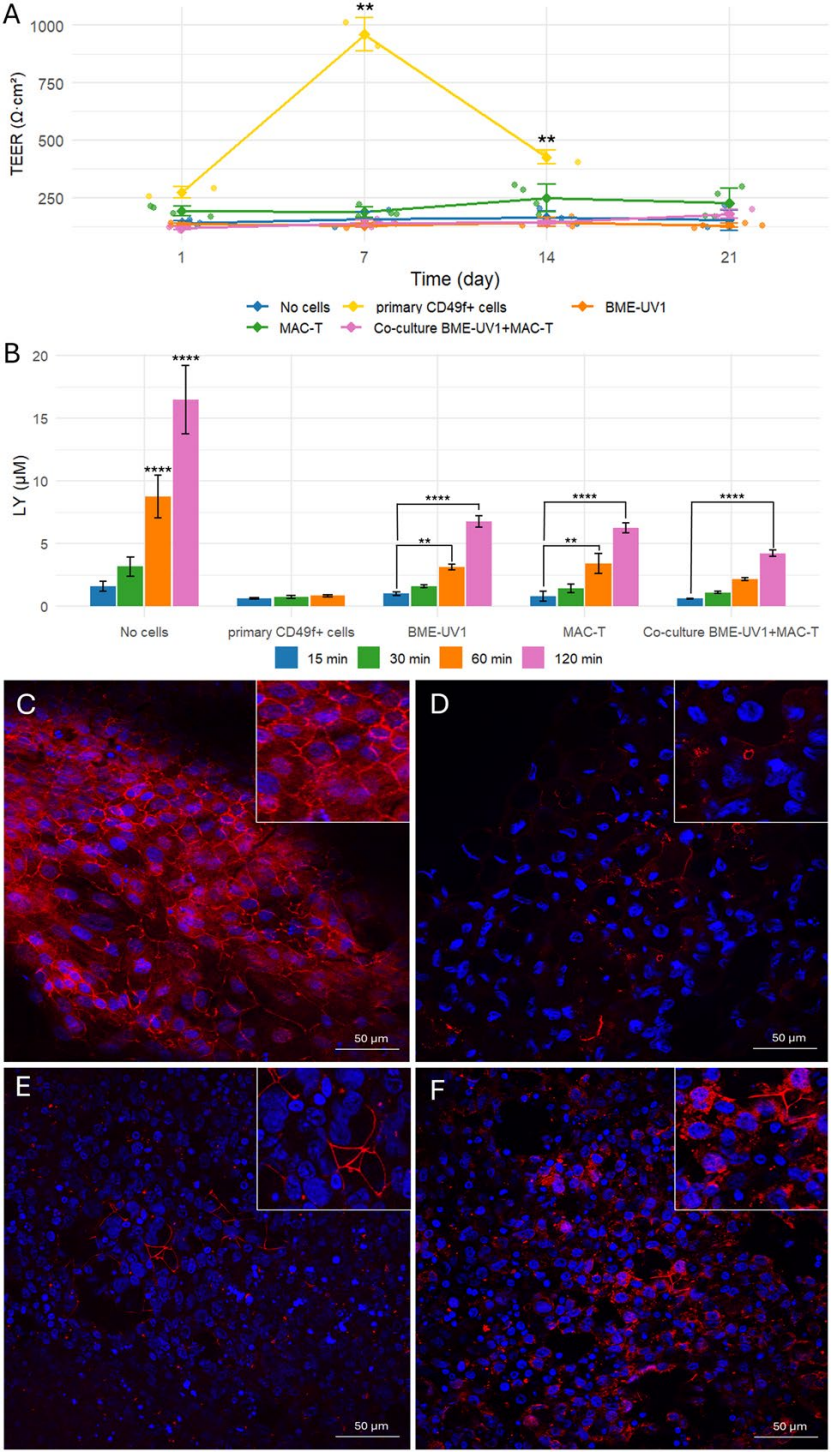
**Evaluation of epithelial barrier formation by primary bMECs and bMEC lines (BME-UV1 and MAC-T) in mono- and co-culture on Transwell® insert.** Kinetics of transepithelial electrical resistance (TEER) for primary CD49f⁺ bMECs, MAC-T, BME-UV1, co-culture and acellular control (**A**). Kinetics of Lucifer Yellow (LY) diffusion on day 14 evaluated during 15, 30, 60 and 120 min, showing LY passage from the upper to the lower compartment (**B**). Data are presented as mean ± SD. Sample sizes: TEER: n = 4 (all groups) except CD49f⁺ primary cells (n = 2); LY: n = 3 (CD49f⁺ primary cells), n = 4 (BME-UV1, MAC-T, co-culture, acellular control). Data are indicated as mean ± standard deviation; p values are represented as ** and ****, indicating p < 0.01, p < 0.0001. Immunofluorescent staining for Zonula Occludens 1 (red) and nuclei (Hoechst 33,342, blue) **(C-F)**. Representative images of CD49f⁺ primary cells on day 9 (**C**), MAC-T (**D**), BME-UV1 (**E**), and their co-culture (**F**) on day 14. Images acquired with an ApoTome™ epifluorescence microscope. A two-time magnification of the tight junction structure is shown in the top right insets.

To assess the epithelial barrier integrity, TEER was measured on days 1 (post-seeding), 7, and 14 for primary bMECs and bMEC lines, and on day 21 only for the latter (Figure 2A), using an Epithelial Voltohmmeter (EVOM3, World Precision Instruments, Friedberg, Germany). In addition, paracellular permeability was evaluated on day 14 using the LY assay (Additional file 2D). In this assay, diffusion of fluorescent LY (200 µM) from the upper to the lower compartment of the Transwell® insert reflects the absence of or compromised tight junction formation required for epithelial barrier function. TEER and LY data were obtained from duplicate measurements in two independent experiments (n = 4) and analyzed by one-way ANOVA followed by Tukey’s post-hoc test using GraphPad Prism. Results were expressed as mean ± standard deviation (SD); *p* < 0.05 was considered significant. Graphical representations were generated in R. In parallel with these functional studies, tight junction formation was investigated by ZO-1 immunofluorescence (IF) after 14 d of culture for bMEC lines and after 9 d of culture for primary bMECs (2 d in proliferation medium to reach confluence, followed by 7 d in differentiation medium). Cells were fixed with 4% paraformaldehyde (PFA) in phosphate-buffered saline (PBS) (VWR, Fontenay-sous-Bois, France), washed twice with PBS, permeabilized with 0.25% Triton X-100 (Merck, Saint-Quentin Fallavier, France), washed again with PBS and blocked for 30 min (min) with 2% bovine serum albumin (BSA). Monolayers were incubated for 1.5 h at 37 °C with primary antibodies diluted in PBS containing 0.2% BSA, followed by two PBS washes and a 1h incubation with the corresponding secondary antibodies (Additional file 2C). Nuclei were counterstained with Hoechst 33,342 (Merck) at 5 µg/mL for 2 min, samples were mounted using 15 μL of ProLong Gold Antifade Mountant (Thermo Fisher, Illkirch, France) and images were acquired with an Apotome™ epifluorescence microscope (Zeiss, Paris, France) using the Zen software (Zeiss).

For primary CD49f⁺ bMECs, TEER values significantly increased from day 1 (273.8 ± 25.5 Ω·cm^2^) to day 7, reaching 960.2 ± 70.7 Ω·cm^2^ (n = 2; *p* < 0.01), followed by a decline by day 14 (425.9 ± 30.4 Ω·cm^2^; *p* < 0.01). However, over time, TEER values of all immortalized cell lines either in mono- or co-culture were comparable to the acellular (no cells) control (152.7 ± 43.8 Ω·cm^2^) except a slight but significant increase for MAC-T at d 21 (*p* < 0.01). For MAC-T, TEER rose modestly from 192.1 ± 20.1 Ω·cm^2^ on day 1 to 226.3 ± 66.7 Ω·cm^2^ on day 21 (*p* < 0.05). For BME-UV1, TEER values remained stable from 134.5 ± 4.8 Ω·cm^2^ on day 1 to 129.3 ± 8.4 Ω·cm^2^ on day 21. In co-culture, TEER values ranged from 119.3 ± 7.1 Ω·cm^2^ on day 1 to 179.5 ± 20.2 Ω·cm^2^ on day 21. No significant differences in TEER profiles over time were observed when bMEC lines were cultured on collagen-coated or Matrigel-coated Transwell® inserts compared to uncoated inserts. Corresponding TEER data are provided in Additional file 3.

LY transfer results (Figure 2B) indicate that in the absence of cells on the Transwell® insert, the fluorescent dye diffused gradually and significantly in the lower compartment from 15 min (1.56 ± 0.39 µM) to 2 h (h) (16.46 ± 2.71 µM; *p* < 0.0001). In contrast to the acellular control, the LY concentration in the lower compartment of the primary cell culture was low and did not significantly diffuse from 15 min (0.59 ± 0.05 µM) to 1 h (0.80 ± 0.08 µM; n = 3). A different time-dependent pattern of LY diffusion was observed for the cell lines in either mono- or co-culture. LY fluorescence diffused significantly in the monocultures from 15 min to 1 h (MAC-T: from 1.01 ± 0.12 µM to 3.39 ± 0.78 µM and BME-UV1: from 0.98 ± 0.14 µM to 3.09 ± 0.24 µM; *p* < 0.01) and up to 2 h (MAC-T: 6.22 ± 0.40 µM; BME-UV1: 6.74 ± 0.46 µM; *p* < 0.0001). In co-culture, the LY diffusion occurred as well, reaching 4.19 ± 0.26 µM at 2 h (*p* < 0.0001). The diffusion assay demonstrated that the cell layers formed by the bMEC lines, in both mono- and co-culture, were permeable, indicating insufficient barrier formation.

To complement these functional barrier assessments, tight junction formation was subsequently evaluated by immunofluorescence staining of ZO-1. Primary CD49f⁺ bMECs showed distinct, smooth, uninterrupted lines at the perimeter of adjacent cells (Figure 2C). In contrast, MAC-T cells showed weak or absent signal at the borders, with increased cytoplasmic staining, and BME-UV1 and their co-culture displayed a loss of the smooth, straight lines, often observed in immature or remodeled junctions (Figure 2D and F). These findings are consistent with conventional 2D monolayer observations (Additional file 4).

Given the limited barrier formation of bMECs observed in 2D as well as on Transwell®, we therefore examined whether 3D culture conditions could promote aspects of epithelial polarization and tight junction organization by assessing BME-UV1 and MAC-T bMEC interactions in a novel mammosphere co-culture model.

### Basal MAC-T cells partially and heterogeneously adhere to BME-UV1 mammospheres in 3D co-culture

**Figure 3:**
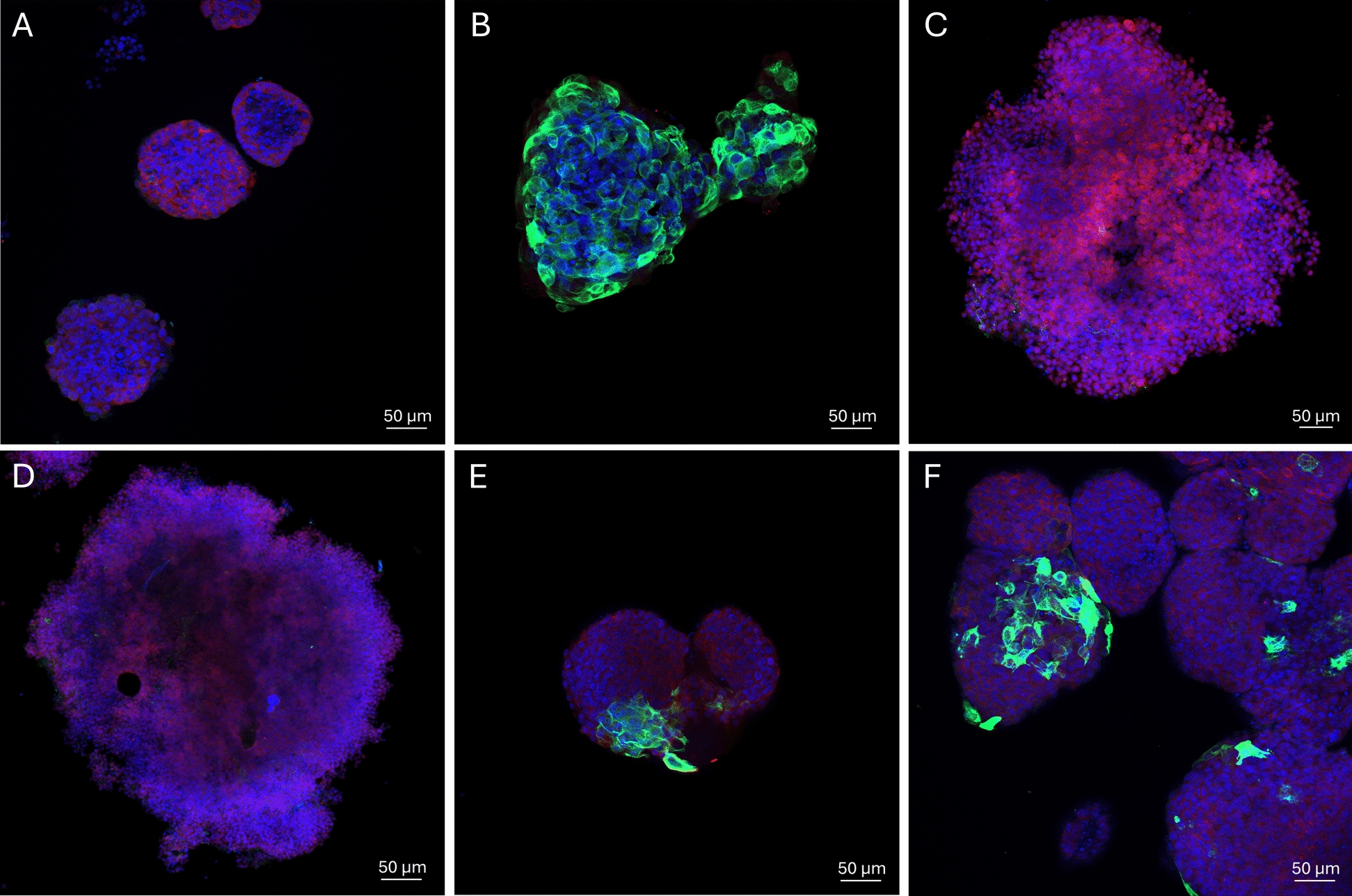
**Immunolabeling of keratins (K) in mammospheres formed by either MAC-T, BME-UV1 or their co-culture after 11 d of culture within various extracellular matrices or in ultra-low attachment (ULA) plates.** Cells were labeled with anti-K14 (green) or anti-K8 (red), and nuclei with Hoechst 33,342 (blue). Monoculture of BME-UV1 (**A**) or MAC-T (**B**) in ULA plates; co-culture of BME-UV1 and MAC-T in collagen type I (**C**); collagen type I supplemented with laminin (**D**); Matrigel® (**E**) and ULA plates (**F**). Images were acquired with an ApoTome™ epifluorescence microscope. A three-time magnification of the cells constituting the mammosphere is shown in the top right insets.

ULA and Matrigel® embedded monoculture of BME-UV1 and MAC-T were initially reproduced according to previously published protocols from our group (Figure 3A and B) [4]. Experimental conditions were adapted to generate BME-UV1 mammospheres, subsequently combined with MAC-T cells in order to evaluate their interactions in different 3D culture environments. In this study, the term “mammosphere” refers to 3D aggregates of bovine mammary epithelial cells formed under non-adherent or ECM-supported conditions, without implying the presence of a fully developed lumen or complete functional polarization. A total of 10,000 BME-UV1 cells were seeded into selected ECM hydrogels or ULA 24-well flat-bottom plates (1.9 cm^2^ surface area). The ECM-based matrices, including Matrigel®, collagen type I from rat tail, collagen type I from rat tail supplemented with mouse laminin (all matrices from Thermo Fisher), and the synthetic xeno-free hydrogel Vitrogel® (TheWell Bioscience, North Brunswick, USA), were all prepared according to the manufacturer’s instructions (Additional file 2E). It should be noted that collagen I and laminin represent selected components of the complex bovine mammary extracellular matrix, which also includes collagen type IV, fibronectin, proteoglycans, glycosaminoglycans, and matrix-associated growth factors. The combination of collagen I and laminin was used to provide defined fibrillar and basement membrane-related cues in a controlled and reproducible in vitro setting, rather than to fully recapitulate the complete bovine extracellular matrix composition. Each condition was tested in two independent experiments. BME-UV1 cells were cultured as mammospheres for 7 d, consisting of 5 d in BME-UV1 proliferation medium followed by 2 d in BME-UV1 differentiation medium. On day 7, MAC-T cells were added to BME-UV1 mammospheres. Mammospheres were recovered from Matrigel® using cell-recovery solution (Thermo Fisher Scientific, France), from collagen-based matrices using collagenase type I CSL-1 (Serlabo, Entraigues, France), and from ULA plates by gentle gravity sedimentation (Additional file 2F). Recovered mammospheres were then centrifuged at 200 x *g* for 10 min, after which the supernatant was removed and the pellet was gently resuspended together with 10,000 MAC-T cells using a pre-coated large-aperture pipette tip. The resulting co-cultures were transferred into the corresponding ECM or ULA plates and maintained for an additional 4 d in differentiation medium consisting of a 1:1 (v/v) mixture of differentiation media. On day 11, phase-contrast images were acquired. For each condition, multiple spheroids (n = 20) were analyzed to measure average spheroid size using FIJI/ImageJ (NIH, USA). Mammosphere diameter measurements were analyzed by one-way ANOVA followed by Tukey’s post-hoc test using GraphPad Prism. Results were expressed as mean ± SD; *p* < 0.05 was considered significant.

For immunofluorescence analysis, mammospheres were recovered from the ECM hydrogels as described above, while those from the ULA condition were collected directly. All samples were transferred to ULA plates and fixed in 4% PFA following the procedures of Dekkers et al. (2019), but without the clearing step [17]. Immunostaining for two selected keratin markers (K8, expressed by luminal cells, and K14, expressed by basal cells) and for the tight junction protein ZO-1 was performed (antibodies listed in the Additional file 2B). All staining steps were performed under a binocular stereomicroscope to minimize mammosphere loss. Fluorescent images of K8 and K14 labeling were acquired with an ApoTome epifluorescence microscope and ZO-1 labeling with a confocal laser scanning microscope, both operated with Zen software (devices and software from Zeiss).

Mammospheres cultivated in ULA conditions were additionally subjected to actin immunostaining for cytoskeletal organization and viability assessments using two complementary approaches. Actin was detected using a primary anti-actin antibody (see Additional file 2B for antibody details). For live imaging of non-fixed spheroids, propidium iodide (Sigma) (0.5 µg/mL) and Hoechst 33,342 (2.5 µg/mL) were added directly into the culture medium prior to fluorescent imaging. In parallel, a Live/Dead viability assay was performed on living mammospheres using the Viobility™ 488/520 fixable dye Kit (Miltenyi Biotec), according to the supplier’s instructions. After staining, mammospheres were gently washed, fixed in 4% PFA, and imaged by fluorescence microscopy.

To enable longitudinal tracking of individual spheroid growth, a dedicated ULA setup was established for kinetic monitoring. For this purpose, 2,500 BME-UV1 cells were seeded into 96-well U-bottom ULA plates and centrifuged at 250 x *g* for 10 min to promote aggregation and ensure the formation of a single centrally positioned spheroid per well. Culture duration, medium composition, and medium changes were identical to those used in the 24-well experiments. On day 7, 2,500 MAC-T cells were added, followed by centrifugation at 250 x *g* for 10 min to facilitate cell contact. Spheroid growth and cell dynamics were monitored by phase-contrast imaging using a BioTek Cytation™ 5 Cell Imaging Multimode Reader integrated with a BioTek BioSpa Live Cell Analysis System (Agilent Technologies, Santa Clara, CA, USA), enabling automated incubation and robotic plate transfer for longitudinal image acquisition. Images were acquired once daily during spheroid formation (day 0–7), every 8 h following MAC-T addition on day 7, and subsequently once daily from day 8 to day 11.

Formation of BME-UV1 spherical cell aggregates was successfully achieved in ULA plates as well as in Matrigel®, collagen type I, and collagen type I supplemented with laminin, whereas no stable aggregate formation was observed in Vitrogel® after 5 d of culture (Additional file 5). Phase-contrast imaging of aggregates embedded in collagen-based matrices was partially hindered by background signal from the matrix. Following the addition of MAC-T cells on day 7, phase-contrast microscopy confirmed the presence of individual MAC-T cells adjacent to the BME-UV1 mammospheres (Additional file 6). In Matrigel®, MAC-T cells were unevenly distributed, leaving empty zones. After 4 additional days of co-culture (day 11), condition-dependent differences in both cellular organization and mammosphere size emerged. In collagen and collagen supplemented with laminin hydrogels, cells appeared to organize into a continuous layer within the matrix, seemingly in proximity to the BME-UV1 mammospheres (Additional file 7A and B). In contrast, in ULA and Matrigel® cultures, cells appeared organized into clusters surrounding the mammospheres (Additional file 7C and D). Mammospheres cultured in collagen-based matrices were significantly larger compared to those formed in Matrigel® or under ULA conditions (*p* < 0.05). Specifically, mean spheroid diameters reached 185 ± 58 µm in collagen and 189 ± 71 µm in collagen supplemented with laminin, whereas mammospheres in Matrigel® and ULA measured 125 ± 23 µm and 131 ± 46 µm, respectively (Additional file 7E).

Immunofluorescence staining of keratins demonstrated distinct co-culture outcomes depending on the matrix environment. In collagen and collagen supplemented with laminin conditions, only K8-positive mammospheres were observed, with no detectable K14 signal, indicating luminal BME-UV1 mammospheres in the absence of surrounding basal MAC-T cells (Figure 3C and D). Although phase-contrast imaging suggested the presence of a continuous cell layer within the matrix in proximity to the BME-UV1 mammospheres under these conditions, this apparent association was not preserved upon immunostaining and could therefore not be confirmed as MAC-T cells. The mammospheres seemed to lack a clear structural definition, with detaching peripheral cells, suggesting structural fragility or peripheral cell aggregation. In contrast, in both the Matrigel®- and ULA-based 3D co-culture conditions, K14-positive MAC-T cells were detected in proximity to the K8-positive BME-UV1 mammospheres. These cells did not organize into a structured network surrounding the BME-UV1 mammosphere (Figure 3E and F). Instead, their distribution was heterogeneous, without forming a coherent basal-like arrangement comparable to that described in vivo. Within these cultures, purely luminal mammospheres composed of K8-positive, K14-negative cells were also observed, indicating variability in the spatial association between MAC-T cells and BME-UV1 mammospheres.

To further investigate the origin of the patch-like cell structures observed by phase-contrast imaging, kinetic phase-contrast imaging was performed under ULA conditions using the BioTek Cytation™ 5 system (Additional file 8). This time-lapse analysis showed that, following the addition of MAC-T cells on day 7, these cells progressively localized and accumulated around the BME-UV1 mammospheres over time, confirming that the observed patch-like structures corresponded to MAC-T cells and providing dynamic support for the static phase-contrast observations.

**Figure 4:**
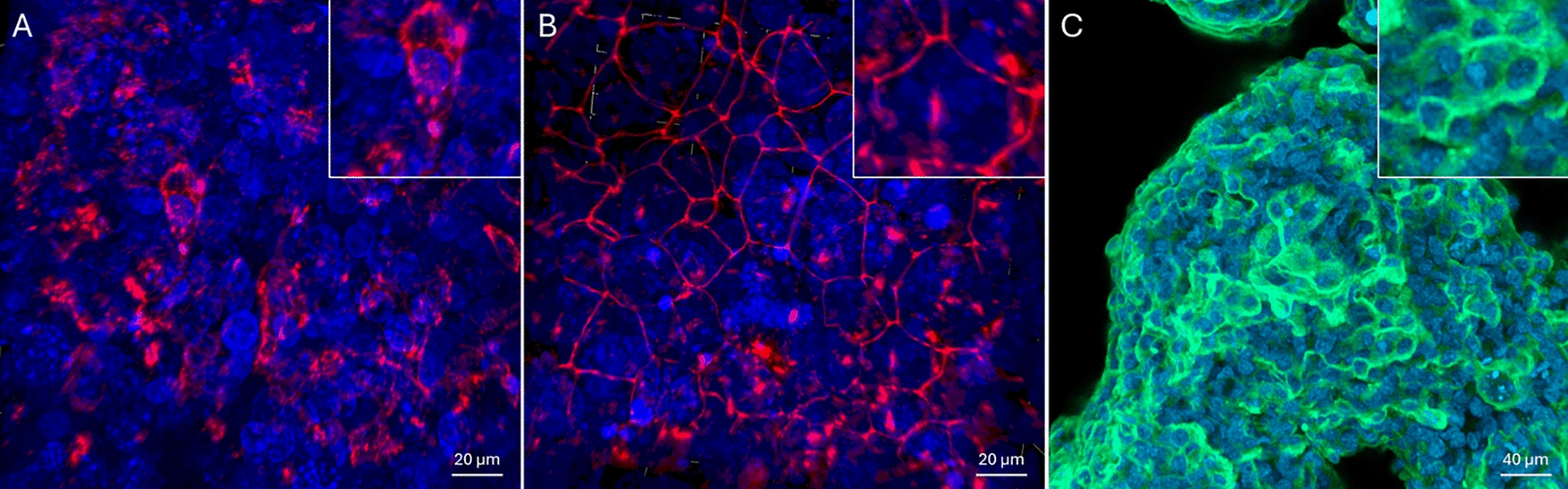
**Organization of tight junctions in co-culture mammospheres after 11 days of culture.** Mammospheres of BME-UV1 co-cultured with MAC-T cells in Matrigel® (**A**) and ultra-low attachment plates (**B**) were labeled with anti-ZO-1 (red) and nuclei with Hoechst 33,342 (blue). In addition, mammospheres cultured under ULA conditions were stained for actin (green) to visualize cytoskeletal organization (**C**). Images were acquired by a confocal laser scanning microscope (CLSM, LSM880, Zeiss) using Zen software (Zeiss). A two-time magnification of the tight junction and actin structures is shown in the top right insets.

To assess epithelial junctional organization within the co-cultured mammospheres, ZO-1 localization was examined by confocal microscopy. Z-stack imaging was performed to evaluate junctional distribution throughout the spheroids (Figure 4A and B), and three-dimensional reconstructions providing an overview of the entire mammospheres is presented in Additional file 9. In both ULA and Matrigel*®*, ZO-1 labeling was visible throughout the mammospheres. In the ULA condition, ZO-1 displayed a sharp, continuous staining pattern outlining cell borders (Figure 4A), indicative of organized junctional localization in epithelial structures, without implying full maturation of tight junction complexes. In contrast, in Matrigel®, ZO-1 displayed a discontinuous and irregular staining pattern, with blurred cell–cell borders and incomplete junctional rings (Figure 4B), compared to the more continuous organization observed under ULA conditions. In parallel, actin organization was assessed in ULA-derived co-cultured mammospheres (Figure 4C). Actin labeling revealed a structured cytoskeletal organization supporting the 3D architecture of the mammospheres, consistent with epithelial organization in the ULA condition.

In addition to differences in junctional organization, phase-contrast suggested the presence of centrally located regions with reduced cellular density within the mammospheres. To further investigate the nature of these regions, viability was assessed in ULA-derived mammospheres. A central accumulation of PI-positive and viability dye-positive staining was observed, indicating reduced cell viability in the core, whereas the outer cell layers remained viable (Additional file 10).

## Discussion

This study aimed to establish a cellular model simulating the in vivo cell–cell interactions and alveolar architecture of the bovine mammary gland. With this aim, we compared the behavior of the bMEC lines BME-UV1 and MAC-T when co-cultured in different 3D conditions, including Transwell® inserts as well as mammosphere systems (ECM-embedded or matrix-free conditions).

First, we evaluated the ability of BME-UV1 and MAC-T to form a mammary epithelial barrier on Transwell® insert. Primary bMECs served as a control to validate the TEER, ZO-1, and LY readouts expected for the establishment of such a functional barrier. For primary bMECs, TEER reached ~ 1000 Ω·cm^2^, and continuous ZO-1  junctional labeling and negligible LY diffusion were observed, in line with previously reported values [18, 19]. In contrast, BME-UV1 and MAC-T mono- as well as co-cultures showed low TEER and only a partial reduction in LY diffusion. For these cell lines, LY diffusion followed a time-dependent pattern, with progressive fluorescence diffusion in the lower compartment. This kinetic pattern was similar to that of the acellular control, indicating that the cell layers formed by the bMEC lines, in both mono- and co-culture, remained permeable and did not establish an effective epithelial barrier. Although LY concentrations in MAC-T and BME-UV1 mono- and co-cultures were lower than in the acellular control, this reduction likely reflected the presence of a physical barrier formed by the cell layer itself rather than the establishment of a functionally tight epithelial barrier. TEER values are highly dependent on cell type and culture conditions. Reported values for bovine mammary epithelial models vary widely, from below 200 Ω·cm^2^ in immortalized lines to over 1000 Ω·cm^2^ in primary or differentiated cell culture models, depending on the degree of junctional maturation and cell polarization [19–22]. In this study, MAC-T and BME-UV1 displayed TEER values around 250 Ω·cm^2^, which may indicate partial monolayer formation. Although TEER of MAC-T cell cultures showed a slight but significant increase over time, the absence of a progressive rise and the lack of a stable plateau suggest that no mature or functional tight epithelial barrier developed. TEER values for BME-UV1 culture and the co-culture remained largely unchanged and within the same order of magnitude as those measured in the acellular control, further supporting their limited barrier-forming capacity under these experimental conditions. Of relevance, comparable low TEER values have been reported for other epithelial cell lines cultured on Transwell®, including an immortalized human intestinal epithelial cell line, highlighting that the ability to establish a tight and well-polarized epithelial barrier in Transwell systems is highly dependent on the cell type and culture conditions, including medium composition, supplements, and matrix properties [23]. Moreover, MAC-T monocultures showed no evidence of junction formation, whereas both BME-UV1 monocultures and BME-UV1/MAC-T co-cultures exhibited partial and discontinuous ZO-1 labeling, consistent with a non-mature junctional organization.

The potential interest of co-culturing BME-UV1 and MAC-T cells was further explored under different 3D conditions, with the additional aim to test ECM alternatives to the most commonly used and controversial Matrigel®. Matrigel® is considered the least animal-friendly option, as it is derived from Engelbreth-Holm-Swarm (EHS) mouse sarcoma. While its exact composition varies between batches, Matrigel® represents a complex and biologically relevant extracellular matrix that has been widely used to reproducibly generate polarized epithelial structures in vitro [24]. To address the ethical and translational limitations associated with tumor-derived matrices, alternative culture systems have been explored, including the matrix-free ULA condition, rat-tail collagen I and synthetic hydrogels such as Vitrogel® [24–26]. Collagen I was also tested in combination with laminin to evaluate whether additional basement-membrane signals could enhance mammosphere development. Indeed, these signals are critical for acinar morphogenesis and epithelial polarity, whereas collagen I alone often yields poorly organized structures [27–29].

Our observations highlight that both mammosphere growth and luminal-basal organization were dependent on the matrix environment. Collagen-based matrices yielded significantly larger mammospheres than Matrigel® or ULA conditions, indicating that scaffold composition influences aggregate growth dynamics. Although mammosphere formation was observed in both collagen and collagen supplemented with laminin conditions, no K14-positive MAC-T cells were detected in association with the BME-UV1 mammospheres by immunofluorescence microscopy. While phase-contrast microscopy suggested the presence of a continuous cellular layer within the collagen-based matrices in proximity to the BME-UV1 mammospheres, this spatial arrangement was not confirmed by K14 immunostaining. Consequently, although both cell lines were cultured within the same matrix environment, a structured luminal-basal co-culture organization could not be demonstrated under collagen or collagen supplemented with laminin conditions. Moreover, both collagen-based matrices hindered clear phase-contrast imaging. This is likely due to their optical density and background interference. Similar limitations have previously been reported by Holland et al. (2007) for bovine mammary progenitors grown in collagen- or laminin-based systems, supporting the notion that these matrices provide limited support for the establishment of organized epithelial structures [30]. As an alternative, Vitrogel® was selected as a fully synthetic, xeno-free hydrogel designed to support various organoid systems. While such alternatives may offer improved standardization or animal-friendliness, detailed information regarding their molecular composition is not fully disclosed. Under our experimental conditions, no mammospheres were obtained in Vitrogel®. This may be related to differences in matrix composition or physicochemical properties that influence cell aggregation, adhesion and epithelial self-organization. However, this observation is specific to our cell lines and culture setup and does not imply that Vitrogel® is generally unsuitable for 3D culture, as other cell types may respond differently depending on their adhesion requirements and matrix interactions. Similarly, Cherne et al. (2021) reported that human gastric organoids cultured in Vitrogel® exhibited slower growth, smaller diameters, and less uniform morphology compared to Matrigel® [31], suggesting that matrix-dependent effects on epithelial organization may occur across systems. Limited transparency in matrix composition may complicate direct comparisons across studies and contribute to variability in reported outcomes in 3D mammary culture systems.

In contrast, morphologically comparable co-cultured mammospheres were obtained under both Matrigel® and ULA conditions. Notably, Matrigel® supported spatial association between luminal and basal cells, whereas such organized co-culture could not be demonstrated in collagen-based matrices. Matrigel® contains basement membrane-associated components such as collagen IV, heparan sulfate proteoglycans, and entactin/nidogen, which are absent from the collagen I-based matrices used in this study. The absence of these components may influence cell–matrix and cell–cell interactions and thereby affect spatial organization within the 3D environment. In ULA and Matrigel® settings, MAC-T cells were detected in proximity to the BME-UV1 mammospheres, displaying an irregular distribution rather than forming a structured basal-like network. Under ULA conditions, kinetic phase-contrast imaging further demonstrated that MAC-T cells progressively localized around pre-formed BME-UV1 mammospheres following their addition, supporting that the peripheral clusters corresponded to active cell association rather than random aggregation. In vivo, myoepithelial cells organize as a network surrounding the luminal epithelial compartment. In our model, MAC-T cells were observed around BME-UV1 mammospheres; however, their distribution remained heterogeneous and did not reproduce such an organized basal arrangement. The spatial pattern observed in our co-culture system therefore only partially reflects luminal-basal epithelial organization in vivo. A possible explanation is that the basal-like phenotype of MAC-T cells, as well as the 4 d co-culture period, may have limited their integration. Nevertheless, 3D co-culture of the bMEC lines to mammospheres was successfully achieved in ULA conditions, which proved to be a suitable alternative culture system to Matrigel®. Comparable myoepithelial-luminal epithelial cell co-cultures have been described with either a commercial human breast cell line [32] or with primary murine mammary epithelial cells [33]. More specifically, Weber-Ouellette et al. (2018) reported the formation of bilayered mammospheres by human luminal MCF-12A and myoepithelial-like Hs 578Bst cells in Matrigel® . In contrast to our findings, their myoepithelial-like cells displayed stellate morphology and expressed α-smooth muscle actin but lacked K14 expression. The MAC-T cell line used in our study did not adopt a stellate morphology and retained K14 expression, suggesting a more basal-like phenotype. This phenotypic difference may have contributed to the inability to recreate a bilayered structure in our model.

Under ULA conditions, mammospheres displayed a sharper co-cultured morphology with continuous ZO-1 organization, suggesting favorable conditions for bovine mammary epithelial cell structuring. Moreover, ULA avoids batch variability, undefined composition, and most importantly, ethical concerns associated with Matrigel®, while being more cost-effective [26]. Direct access to mammospheres without a matrix recovery step represents another advantage of ULA. Beyond these advantages, ULA also provides a robust, reproducible, and completely animal-free platform suitable for high-throughput drug testing, as demonstrated in original studies [34–36] and recently confirmed for human mammary cell lines cultured in ULA systems [37]. Moreover, we showed that ULA culture supported the formation of a stronger barrier within the BME-UV1 mammospheres than Matrigel®. Central tight junction localization has previously been reported in BME-UV1 spheroids cultured in Matrigel®, where ZO-1 labeling was observed predominantly in the spheroid center [13]. In contrast, under ULA conditions, ZO-1 was distributed in a continuous pattern throughout the mammosphere, and this organization was accompanied by a structured actin cytoskeleton, supporting the three-dimensional epithelial architecture of the mammospheres. However, in dense 3D cultures, centrally located cavities were observed. They do not necessarily indicate the formation of a physiologically organized luminal compartment. Such regions may arise from diffusion-limited conditions, resulting in reduced nutrient and oxygen availability and subsequent cell death in the spheroid core. Similar necrotic core formation has been reported in 3D spheroid and organoid models [38, 39]. This effect is known to be size-dependent, with the extent of central necrosis increasing progressively as spheroid diameter exceeds a critical threshold between 200 and 500 µm, depending on cell type and metabolic demand [34]. In our study, the centrally localized PI-positive staining and Live/Dead viability staining, combined with preserved peripheral viability, support the interpretation of a necrotic core rather than lumen formation. Together, these findings suggest that centrally located regions described in previous mammosphere studies may, at least in part, reflect necrotic cell loss rather than bona fide lumen formation. Importantly, the mammospheres analyzed in this study did not reach a fully mature alveolar stage, and the observed ZO-1 distribution therefore reflects junctional organization at this developmental stage rather than complete apico-basal polarization of mature alveoli.

The fundamental improvement of this co-culture system lies in re-establishing the luminal-basal epithelial hierarchy within a 3D context. However, our conclusions are primarily based on structural and viability-based readouts assessing epithelial organization. While these parameters provide insight into junctional integrity and cellular arrangement, additional functional evaluations would further strengthen the model. Quantitative permeability assays in 3D could further refine the assessment of barrier function, as demonstrated in murine intestinal organoids using diffusion of fluorescent tracers combined with 3D imaging [40]. Moreover, evaluation of downstream functional outputs, such as differentiation-associated markers or contractile responses, would help determine whether this architectural organization translates into functional epithelial maturation. Further enhancement of physiological relevance could include the establishment of epithelial polarity, intercellular communication, and synthesis of milk components such as β-casein, lactose, and triglycerides. Integration of immune components would also add value, given their central role in mastitis pathogenesis and host response to infection. Together, these approaches represent important next steps in further model refinement.

In the context of future bovine mastitis research, an in vitro cell model with accessible luminal cells is desirable, since therapeutic agents are administered intramammarily and would primarily target luminal cells. An inverted model of MAC-T mammospheres surrounded by BME-UV1 cells would be of interest for the study of drug delivery and barrier penetration. Although such a configuration would not recapitulate the in vivo alveolar organization, it would retain the co-culture context and thereby preserve cell–cell interactions between luminal and basal epithelial populations. This multicellular setting would allow evaluation of how intramammary administered compounds or pathogens affect luminal cells as the primary point of contact, while simultaneously assessing the consequences on epithelial integrity and intercellular crosstalk within the combined system.

In conclusion, the bovine BME-UV1 and MAC-T cell lines were compatible in co-culture but failed to establish a functional epithelial barrier on Transwell® inserts. In 3D culture, BME-UV1 mammospheres developed tight junctions, while MAC-T cells were observed in spatial association with the mammospheres under Matrigel® and ULA conditions without forming an organized basal-like network. Although full alveolar reconstruction was not achieved, this study provides initial insights into luminal-basal co-culture, spheroid formation, and matrix-dependent organization in bMECs. Future investigations should address whether the observed limitations relate to matrix incompatibility, absence of specific adhesion cues, or active exclusion mechanisms. Among the 3D culture support tested, ULA-based co-cultured mammospheres emerged as the most reproducible, animal-free and promising model to investigate mammary epithelial organization, mastitis pathogenesis, and preclinical drug and antimicrobial testing.

## Supplementary Information

**Additional file 1. Determination of optimal medium for co-culture of BME-UV1 and MAC-T cells.** 2D co-cultureof BME-UV1 and MAC-T cells using MAC-T **(A)** and BME-UV1 **(B)** in proliferation medium (2 days) followed by differentiation medium (3 days). 2D co-culture of BME-UV1 with MAC-T in a 1:1 (v/v) mix of BME-UV1 and MAC-Tdifferentiation medium **(C)**. Co-culture under the same 1:1 differentiation medium with additional Ki67 labeling (pink) toassess cell proliferation **(D)**. Monoculture of BME-UV1** (E)** and MAC-T** (F)** in a 1:1 (v/v) mix of BME-UV1 and MAC-Tdifferentiation media. MAC-T (basal) cells were labeled with anti-keratin (K) 14 (green) (a-f), BME-UV1 (luminal) withanti-K7 (red) (a-c and e-f) or anti-K19 (red) (d) and nuclei with Hoechst 33342 (blue) (a-f). Images were acquired byfluorescence microscopy using an ApoTome™ epifluorescence microscope (Zeiss).

**Additional file 2. Supplemental Materials and Methods.**

**Additional file 3. Kinetics of transepithelial electrical resistance (TEER) of MAC-T and BME-UV cells cultured on coated Transwell® inserts.** TEER measurements of MAC-T and BME-UV1 cells cultured on collagen-coated andMatrigel-coated Transwell® inserts. TEER kinetics were assessed under identical experimental conditions. Data arepresented as mean ± standard deviation. Sample sizes: n = 4 for all conditions except for timepoint 21h (n = 2).

**Additional file 4. Zonula Occludens 1 (ZO-1) labeling of MAC-T and BME-UV1 in monolayer (2D) culture.** Monolayers of MAC-T **(A)** and BME-UV1 **(B)** cultured in a 1:1 mix of BME-UV1 and MAC-T differentiation medium for14 days were labeled with anti-Zonula Occludens 1 (ZO-1) (red) and nuclei with Hoechst 33342 (blue) and imaged bymicroscopy using an ApoTome™ epifluorescence microscope (Zeiss).

**Additional file 5. Phase-contrast imaging of BME-UV1 mammospheres after 5 days in different 3D culture conditions.** BME-UV1 were cultured during 5 days with proliferation medium in matrix-free ultra-low attachmentplastic **(A)**, or in the presence of extracellular matrix: Matrigel®** (B)**, collagen type I hydrogel **(C)**, collagen type I andlaminin hydrogel **(D)**, and Vitrogel® **(E)**. Images were acquired using a phase-contrast microscope (Zeiss).

**Additional file 6. Phase-contrast imaging of BME-UV1 mammospheres after 7 days in different 3D culture conditions.** BME-UV1 were cultured for 5 days in proliferation medium, followed by 2 days in differentiation medium,after which MAC-T cells were added and co-cultured in differentiation medium consisting of a 1:1 (v/v) mixture of therespective differentiation media. Cells were grown in different conditions: matrix-free ultra-low attachment plastic** (A)**,or in the presence of extracellular matrix: Matrigel® **(B)**, collagen type I hydrogel **(C)**, and collagen type I and lamininhydrogel **(D)**. Images were acquired using a phase-contrast microscope (Zeiss).

**Additional file 7. Phase-contrast imaging and mean size quantification of mammospheres from co-culturedBME-UV1 and MAC-T cells after 11 days in different 3D culture conditions.** Cells were grown under different conditions: collagen type I hydrogel **(A)**, collagen type I supplemented with laminin hydrogel **(B)**, matrix-free ultra-lowattachment plastic **(C)**, and Matrigel® **(D)**. Continuous cell layers within the matrix are indicated with green arrows.Clusters of cells surrounding the BME-UV1 mammospheres are indicated with red arrows. Images were acquiredusing a phase-contrast microscope (Zeiss). Mammosphere diameter was quantified** (E)**. Data are presented as mean± standard deviation; different letters (a-c) indicate statistically significant differences between culture conditions (*p* <0.05).

**Additional file 8. Kinetic phase-contrast imaging from seeding to day 11 of a single mammosphere from cocultured BME-UV1 and MAC-T cells. ** BME-UV1 mammosphere formation was monitored at 0, 8 and, 16 h **(A-C)**,and at days 3 **(D)** and 4 **(E)** when cultured in proliferation medium. MAC-T cells were added to the BME-UV1mammosphere on day 7** (F)** and co-cultured in differentiation medium consisting of a 1:1 (v/v) mixture of therespective differentiation media. The co-cultured mammosphere was subsequently visualized 8 h **(G)** and 16 h **(H)**after MAC-T addition, and further monitored on days 8** (I)**, 9 **(J)**, 10 **(K)**, and 11 **(L)**. Red arrows indicate MAC-T cells.Images were acquired using a BioTek Cytation 5 Cell Imaging Multimode Reader (Agilent Technologies).

**Additional file 9. Zonula Occludens 1 (ZO-1) labeling of mammospheres from co-cultured BME-UV1 and MAC-T cells after 11 days of culture. **Mammosphere from co-cultured BME-UV1 and MAC-T cells in ultra-low attachmentplates** (A) **and Matrigel® **(B)** were labeled with anti-ZO-1 (red) and nuclei with Hoechst 33342 (blue). Z-stack imagingand three-dimensional reconstructions were performed to provide an overview of ZO-1 distribution throughout theentire mammosphere. Images were acquired by confocal laser scanning microscope (CLSM, LSM880, Zeiss) usingZen software (Zeiss).

**Additional file 10. Viability assessment of single mammosphere from co-cultured BME-UV1 and MAC-T cellsin Ultra-Low-Attachment (ULA) conditions after 11 days of culture.**Mammosphere from co-cultured BME-UV1and MAC-T cells in ultra-low attachment plates at 11 days of culture were assessed for cell viability using twocomplementary approaches: Live imaging of a non-fixed mammosphere stained with propidium iodide (PI; red) andHoechst 33342 (blue) **(A)**; Live/Dead viability assay performed on a second mammosphere using the Viability™488/520 Fixable Dye (green) in combination with Hoechst 33342 (blue) **(B)**. Images were acquired by fluorescencemicroscopy using a THUNDER epifluorescence microscope (Leica).

## Data Availability

The datasets used and/or analyzed during the current study are available from the corresponding author on reasonable request.
