# Supplementary material for: From inserts to 3D spheroids: MAC-T and BME-UV1 co-culture models for in vitro reconstruction of the bovine mammary epithelial architecture
Source: Vet Res. 2026 Jul 3;57:119. doi: 10.1186/s13567-026-01763-5 (PMC13332615; doi:10.1186/s13567-026-01763-5)
Supplement: Supplementary file 1 — Additional file 1. Determination of optimal medium for co-culture of BME-UV1 and MAC-T cells. 2D co-cultureof BME-UV1 and MAC-T cells using MAC-T (A) and BME-UV1 (B) in proliferation medium (2 days) followed by differentiation medium (3 days). 2D co-culture of BME-UV1 with MAC-T in a 1:1 (v/v) mix of BME-UV1 and MAC-Tdifferentiation medium (C). Co-culture under the same 1:1 differentiation medium with additional Ki67 labeling (pink) toassess cell proliferation (D). Monoculture of BME-UV1 (E) and MAC-T (F) in a 1:1 (v/v) mix of BME-UV1 and MAC-Tdifferentiation media. MAC-T (basal) cells were labeled with anti-keratin (K) 14 (green) (a-f), BME-UV1 (luminal) withanti-K7 (red) (a-c and e-f) or anti-K19 (red) (d) and nuclei with Hoechst 33342 (blue) (a-f). Images were acquired byfluorescence microscopy using an ApoTome™ epifluorescence microscope (Zeiss). [file 13567_2026_1763_MOESM1_ESM.docx]

### Additional file 1. Determination of optimal medium for co-culture of BME-UV1 and MAC-T cells


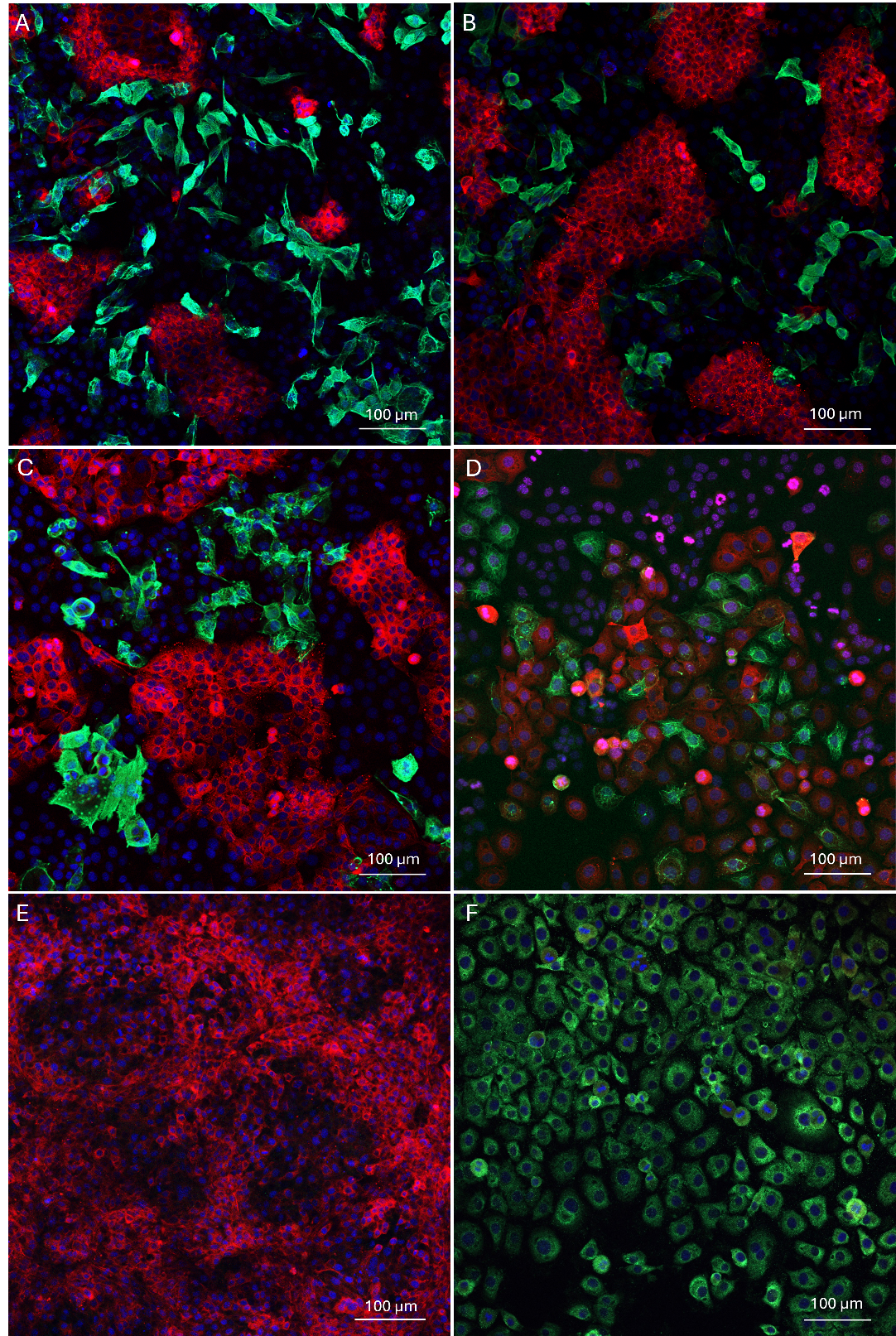


2D co-culture of BME-UV1 and MAC-T cells using MAC-T **(A)** and BME-UV1 **(B)** in proliferation medium (2 days) followed by differentiation medium (3 days). 2D co-culture of BME-UV1 with MAC-T in a 1:1 (v/v) mix of BME-UV1 and MAC-T differentiation medium **(C).** Co-culture under the same 1:1 differentiation medium with additional Ki67 labeling (pink) to assess cell proliferation **(D)**. Monoculture of BME-UV1 **(E)** and MAC-T **(F)** in a 1:1 (v/v) mix of BME-UV1 and MAC-T differentiation media. MAC-T (basal) cells were labeled with anti-keratin (K) 14 (green) (a-f), BME-UV1 (luminal) with anti-K7 (red) (a-c and e-f) or anti-K19 (red) (d) and nuclei with Hoechst 33342 (blue) (a-f). Images were acquired by fluorescence microscopy using an ApoTome™ epifluorescence microscope (Zeiss).
