# Supplementary material for: From inserts to 3D spheroids: MAC-T and BME-UV1 co-culture models for in vitro reconstruction of the bovine mammary epithelial architecture
Source: Vet Res. 2026 Jul 3;57:119. doi: 10.1186/s13567-026-01763-5 (PMC13332615; doi:10.1186/s13567-026-01763-5)
Supplement: Supplementary file 2 — Additional file 2. Supplemental Materials and Methods. [file 13567_2026_1763_MOESM2_ESM.docx]

# Additional file 2. Supplemental Materials and Methods

1. **Culture media**

*Chemicals, cell culture reagents and materials.* Dulbecco’s modified Eagle’s medium (DMEM)/F12 GlutaMAX (11559726), RPMI-1640 (11554516), NCTC 135 (11514486), DMEM High Glucose GlutaMAX (32430-027), bovine pituitary extract (11568866), fetal bovine serum (FBS; 17479633), Phosphate-buffered saline (PBS; 14200-059), Antibiotics (Penicillin-Streptomycin; 11528876) and 10× PBS (14190-094) were obtained from Thermo Fisher Scientific (Illkirch, France). Hydrocortisone (H0396), α-lactose monohydrate (L2643), L-glutathione (G6013), lactalbumin enzymatic hydrolysate (L9010), ovine prolactin (L6520), dexamethasone (D4902-25MG), water-soluble progesterone (P7556), bovine holo-transferrin (T1283), L-ascorbic acid (A4403), bovine insulin (I0516-5ML), Epidermal Growth Factor (EGF, E9644-.2MG), Insulin Growth Factor 1 (IGF-1, I3769-50UG), Insulin-Transferrin-Selenium (ITS 100X, I3146-5ML) and 0.25% trypsin/EDTA (25200-072) reagents were obtained from Sigma Aldrich Chimie (St. Quentin Fallavier, France) now Merck (Molsheim, France). Dual-chamber counting slides (145-0011) with trypan blue (145-0013) were obtained from Bio-Rad (Marnes-la-Coquette, France).

*Cell lines and culture conditions.* The original bovine mammary epithelial cell (bMEC) line (MAC-T) was purchased at Nexia Biotechnologies (Quebec, Canada). MAC-T cells were grown in a proliferation medium consisting of high-glucose DMEM supplemented with 2.5% heat-inactivated FBS, 5µg/mL of bovine insulin and 1µg/mL hydrocortisone. Cells were maintained at 37°C in a 95% humidified atmosphere containing 5% CO_2_ and passaged twice a week. The bovine mammary epithelial BME-UV1 cell line was established by Professor Boris Zavizion (Department of Animal and Food Sciences, University of Vermont, USA). BME-UV1 cells used in this study were originally purchased from the Istituto Zooprofilattico Sperimentale della Lombardia e dell’Emilia Romagna (Brescia, Italy). In this study, aliquots of cryopreserved cells after four passages from the original stock were used. After thawing of cryopreserved cells, the cells were transferred to proliferation medium consisting of DMEM/F-12, RPMI-1640 and NCTC 135 mixed at a 5:3:2 ratio (by volume), supplemented with 0.1% alpha-lactose monohydrate, 1.2 mM L-glutathione, 1 µg/mL bovine insulin, 5 µg/mL bovine holo-transferrin, 1 µg/mL hydrocortisone, 10 µg/mL L-ascorbic acid, 0.5 µg/mL water-soluble progesterone, 0.1% Lactalbumin enzymatic hydrolysate, 10% (vol/vol) heat-inactivated FBS in an atmosphere of 5% CO2/95% humidified air at 37°C. After 3 hours, the medium was replaced by proliferation medium containing 2.5% FBS. Sorted bovine primary mammary cells were grown in Mammary Gland Epithelial Proliferation (MGEP) medium constituted of high-glucose DMEM-F12 supplemented with 10% heat-inactivated FBS, 50 U/ml of antibiotics, 10 ng/mL of EGF, 10 ng/mL of IGF-1, 5µg/mL of hydrocortisone and 0.25X of ITS corresponding to 2.5 µg/ml of Insulin-1.375 µg/ml of Transferrin and 1.25 ng/ml of Selenium.

*Pre-experimental treatment.* MAC-T and BME-UV1 cell lines were thawed and cultured in their respective proliferation media until confluence. After three to four passages over a period of two weeks, the experiment was initiated. Cells were inspected under the microscope to verify their confluence and typical morphology and subsequently harvested using 0.25% trypsin-EDTA for downstream cell viability and proliferation analyses. After trypsinization, cells were stained with Trypan Blue 0.4% (2553352; Bio-Rad) at a final concentration of 0.2% and counted using TC20 Automated cell counter (Bio-Rad). All cells received the same pre-experimental treatment. For the mammosphere assays with and without extracellular matrix (ECM), 10,000 cells per well were seeded in 24-well plate (well size 1.9 cm^2^), while for the Transwell® insert (0.4 μm pore size, 1.1 cm^2^) assay 1 × 10^5^ cells were plated. Sorted bovine primary cells were seeded on Transwell® insert at 1 × 10^5^ cells and cultured until confluency in MGEP medium. For coating Transwell® insert with rat collagen type I or Matrigel®, 250µl of matrix diluted (1:2) with cold DMEM was added on each insert. Plates with coated inserts were placed at 37°C in an incubator for 15 min following by a washing step of inserts with 400 µL of proliferation medium before seeding cells. Media were replaced every two day. Both cell lines were tested for mycoplasma and other bacterial contamination using a PlasmoTestTM mycoplasma detection kit (Invivogen, San Diego, USA) and were found negative for contamination.

*Differentiation conditions*. Depending on the assays, functional differentiation of MAC-T and BME-UV1 cells was induced at the appropriate time point by supplementing the culture medium with 5 µg/mL ovine prolactin, 5 µg/mL dexamethasone and 0.1 % bovine pituitary extract (BPE). In all differentiation media, the FBS concentration was reduced from 2.5% to 0.1%. For sorted bovine primary cells cultured on Transwell® insert, proliferation medium (MGEP) was replaced at the time of cellular confluency (day 3) with differentiation medium consisting of MGEP at 5 µg/mL ovine prolactin, 0.1% FBS and BPE in concentration.

1. **Antibodies**

List of antibodies used in this study, including target name, provider, reference number, application, and dilution conditions for immunostaining (IF) experiments.

| Name (target) | Provider | Reference | Application | Dilution used |
| --- | --- | --- | --- | --- |
| Anti-CD49f biotin conjugated | Miltenyi Biotec | 130-097-243 | Sorting | 1 :10 |
| Anti-biotin-PE conjugated | Miltenyi Biotec | 130-097-243 | Sorting | 1 :10 |
| Anti-K14 | Santa Cruz Biotechnology | SC17104 | IF | 1 :100 |
| Anti-K7 | Santa Cruz Biotechnology | SC70936 | IF | 1 :100 |
| Anti-K8 | Santa Cruz Biotechnology | SC134484 | IF | 1 :100 |
| Anti-K19 | Novocastra (Leica) | NCL-CK19 | IF | 1:200 |
| Anti-ZO1 Alexa Fluor 594 conjugated | Invitrogen | 339194 | IF | 1 :100 |
| Anti-Ki67 | Abcam | Ab15580 | IF | 1:200 |
| Anti-β-Actine | Merck | A5441 | IF | 1 :200 |
| Anti-mouse Alexa Fluor 568 | Thermo Fisher Scientific | A11031 | IF | 1 :500 |
| Anti-goat Alexa Fluor 488 | Thermo Fisher Scientific | A11055 | IF | 1 :500 |
| Anti-rabbit Alexa Fluor 568 | Thermo Fisher Scientific | 406414 | IF | 1 :500 |
| Anti-mouse FITC | Fisher Scientific | F2761 | IF | 1 :500 |
| Anti-rabbit Alexa Fluor 647 | Biolegend | 406414 | IF | 1 :500 |

1. **Fluorescence-Activated Cell Sorting (FACS)**

Primary mammary epithelial cells were sorted by an immunomagnetic separation technique using MS column (130-042-201, Miltenyi Biotec, Paris, France), anti-biotin microbeads (130-090-485, Miltenyi Biotec) and dead cell removal kit (130-090-101, Miltenyi Biotec) according to the manufacturer’s instructions.

1. **Lucifer yellow (LY) assay**

In each plate containing Transwell® insert, the culture medium of the lower and upper compartments was removed and the cell monolayers were washed with 1 mL of HBSS (H8264, Sigma-Aldrich, France). HBSS (1 mL) was added to the lower compartment and LY (L0144-25mg, Merck, France) at 200 μM in HBSS was added to the upper compartment containing cells or not (acellular control). The monolayers were incubated in the dark at 37 °C, and 100 μL of the medium in the lower compartment was removed at 15, 30, 60, and 120 min and replaced by 100 μL of “fresh” HBSS. The fluorescence was measured at an emission wavelength of 540 nm (excitation at 428 nm) with a microplate reader (Varioskan Lux, Thermo Fisher Scientific, France). The LY concentration (μM) was determined from a standard curve (0 to 200 μM). Experiments were performed in triplicate.

1. **3D reagents and consumables**

Matrigel® (11543620), Rat tail collagen type I (A1048301), Mouse Corning™ Ultrapure Laminin (10152421) and ultra-low attachment (ULA; 174930) plates were purchased from Thermo Fisher Scientific and Vitrogel® 3D High Concentration (#TWG001) from TheWell Bioscience (North Brunswick, NJ, USA).

1. **Recovery of mammospheres from extracellular matrices (ECM)**

To avoid sticking of mammospheres to plastic, tips and microtubes were extemporaneously pre-coated with sterile 5% FBS in PBS.

For Matrigel® and collagen-embedded mammospheres, medium was removed and each 3D gel droplet (referred to as a dome) was rinsed with 1 mL of warm sterile PBS. For the Matrigel®-embedded mammospheres, the plate was placed on ice. 1 mL of Cell recovery solution (354253, Thermo Fisher Scientific, France) was added to each well containing a dome. The plate was incubated at 4°C for 30 min. Gentle pipetting was done to help dissolve the Matrigel® using a pre-coated large-aperture tip. For the not fully dissolved Matrigel® dome, the plate was replaced for 10 min at 4°C. For the collagen-embedded mammospheres, 500 µL of collagenase (Collagenase type I CSL-1 of 282U/mg, Serlabo, Entraigues, France) at 1 mg/mL was added to each well containing a dome. The plate was incubated at 37°C for 20 min. Gentle pipetting was done to help dissolve the collagen using a pre-coated large-aperture tip. For the not fully dissolved collagen dome, the plate was replaced for an extra 10 min at 37°C.

After complete dissolution of the ECM, mammospheres were transferred to a pre-coated 2 mL microtube and centrifuged for 10 min at 200 g at 4°C. The supernatant was removed and mammospheres were washed with 1 mL of cold sterile PBS, then centrifuged again.

For recovery of mammospheres from ULA plates, plates were tilted at a 45° angle and left for 5 to 10 min to allow gravity sedimentation of the mammospheres (gravity step). Approximately 800 µL of medium was then carefully aspirated, leaving 100 to 200 µL per well. Mammospheres were washed with 1 mL of PBS, followed by a second gravity step. After adding 1 mL of sterile PBS, the suspension containing mammospheres was transferred into pre-coated 2 mL microtubes and centrifuged for 10 min at 200 g, after which the supernatant was discarded.
