# Supplementary material for: From inserts to 3D spheroids: MAC-T and BME-UV1 co-culture models for in vitro reconstruction of the bovine mammary epithelial architecture
Source: Vet Res. 2026 Jul 3;57:119. doi: 10.1186/s13567-026-01763-5 (PMC13332615; doi:10.1186/s13567-026-01763-5)
Supplement: Supplementary file 3 — Additional file 3. Kinetics of transepithelial electrical resistance (TEER) of MAC-T and BME-UV cells cultured on coated Transwell® inserts. TEER measurements of MAC-T and BME-UV1 cells cultured on collagen-coated andMatrigel-coated Transwell® inserts. TEER kinetics were assessed under identical experimental conditions. Data arepresented as mean ± standard deviation. Sample sizes: n = 4 for all conditions except for timepoint 21h (n = 2). [file 13567_2026_1763_MOESM3_ESM.docx]

### Additional file 3: Kinetics of transepithelial electrical resistance (TEER) of MAC-T and BME-UV1 cells cultured on coated Transwell® inserts


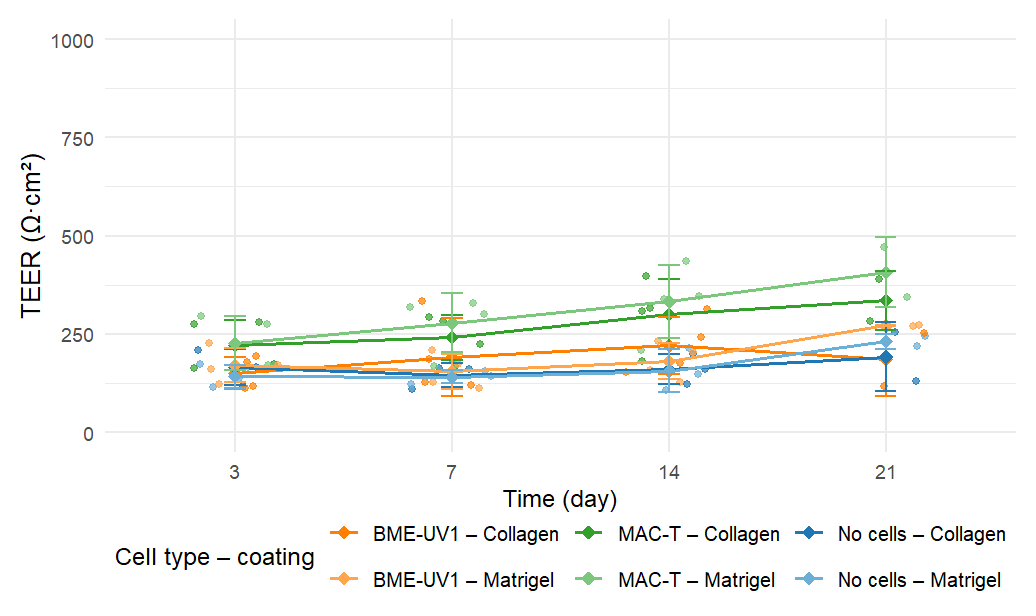


TEER measurements of MAC-T and BME-UV1 cells cultured on collagen-coated and Matrigel-coated Transwell® inserts. TEER kinetics were assessed under identical experimental conditions. Data are presented as mean ± standard deviation. Sample sizes: n = 4 for all conditions except for timepoint 21h (n = 2).
