# Supplementary material for: From inserts to 3D spheroids: MAC-T and BME-UV1 co-culture models for in vitro reconstruction of the bovine mammary epithelial architecture
Source: Vet Res. 2026 Jul 3;57:119. doi: 10.1186/s13567-026-01763-5 (PMC13332615; doi:10.1186/s13567-026-01763-5)
Supplement: Supplementary file 4 — Additional file 4. Zonula Occludens 1 (ZO-1) labeling of MAC-T and BME-UV1 in monolayer (2D) culture. Monolayers of MAC-T (A) and BME-UV1 (B) cultured in a 1:1 mix of BME-UV1 and MAC-T differentiation medium for14 days were labeled with anti-Zonula Occludens 1 (ZO-1) (red) and nuclei with Hoechst 33342 (blue) and imaged bymicroscopy using an ApoTome™ epifluorescence microscope (Zeiss). [file 13567_2026_1763_MOESM4_ESM.docx]

### Additional file 4. Zonula Occludens 1 (ZO-1) labeling of MAC-T and BME-UV1 in monolayer (2D) culture


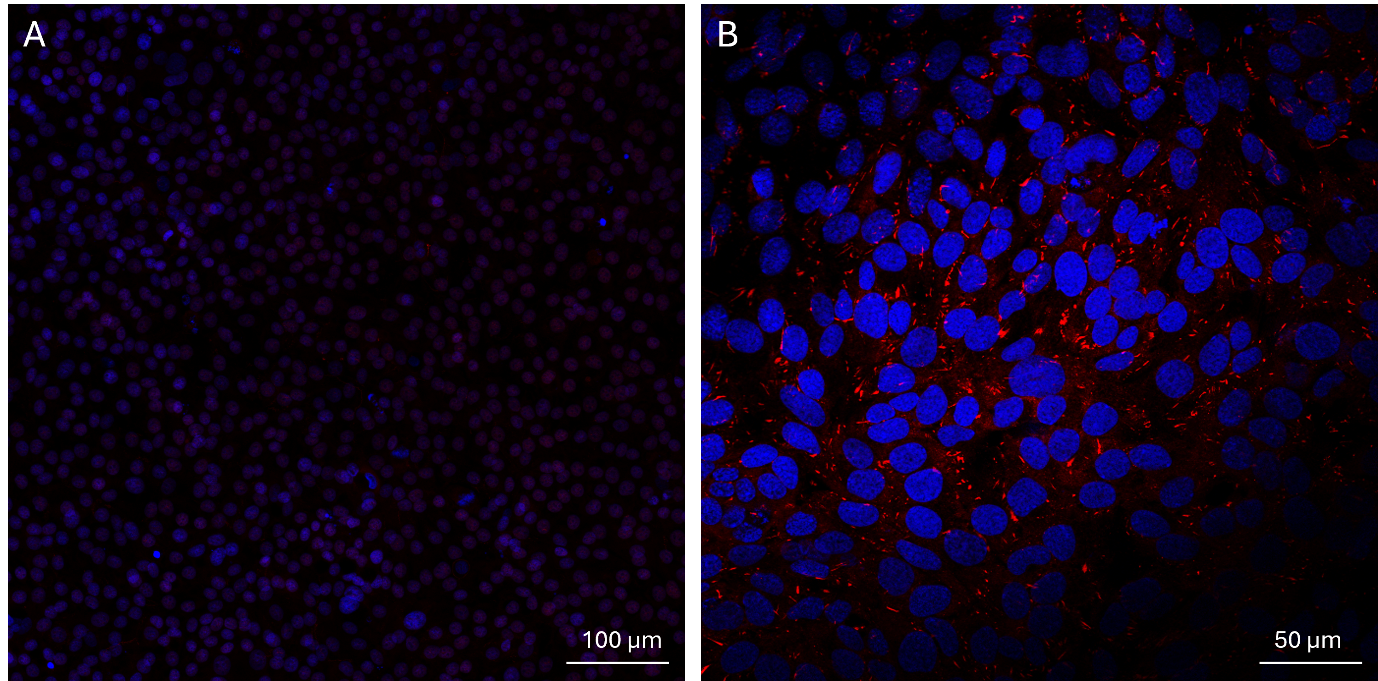


Monolayers of MAC-T **(A)** and BME-UV1 **(B)** cultured in a 1:1 mix of BME-UV1 and MAC-T differentiation medium for 14 days were labeled with anti-Zonula Occludens 1 (ZO-1) (red) and nuclei with Hoechst 33342 (blue) and imaged by microscopy using an ApoTome™ epifluorescence microscope (Zeiss).
