# Supplementary material for: From inserts to 3D spheroids: MAC-T and BME-UV1 co-culture models for in vitro reconstruction of the bovine mammary epithelial architecture
Source: Vet Res. 2026 Jul 3;57:119. doi: 10.1186/s13567-026-01763-5 (PMC13332615; doi:10.1186/s13567-026-01763-5)
Supplement: Supplementary file 5 — Additional file 5. Phase-contrast imaging of BME-UV1 mammospheres after 5 days in different 3D culture conditions. BME-UV1 were cultured during 5 days with proliferation medium in matrix-free ultra-low attachmentplastic (A), or in the presence of extracellular matrix: Matrigel® (B), collagen type I hydrogel (C), collagen type I andlaminin hydrogel (D), and Vitrogel® (E). Images were acquired using a phase-contrast microscope (Zeiss). [file 13567_2026_1763_MOESM5_ESM.docx]

### Additional file 5. Phase-contrast imaging of BME-UV1 mammospheres after 5 days in different 3D culture conditions


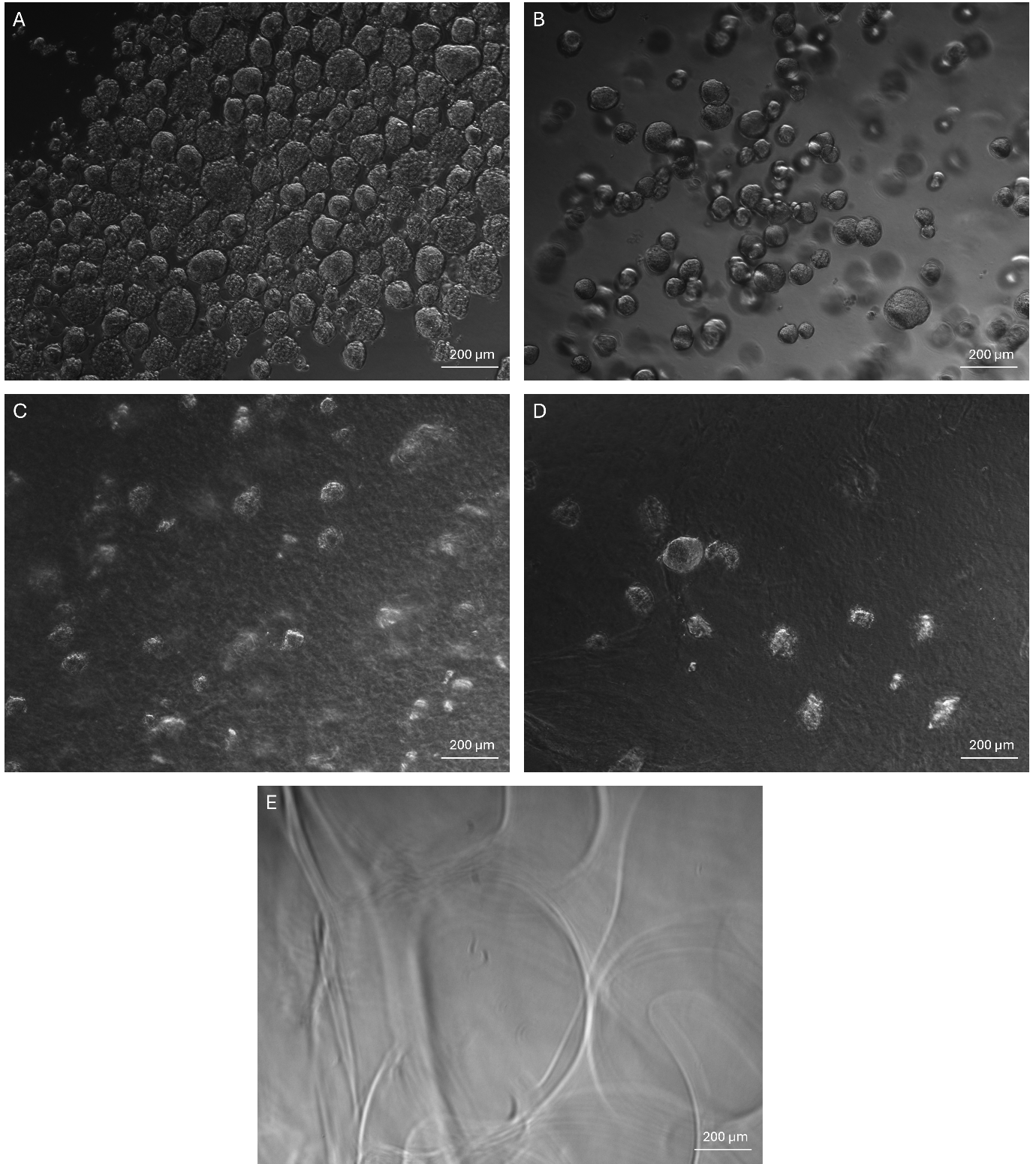


BME-UV1 were cultured during 5 days with proliferation medium in matrix-free ultra-low attachment plastic **(A)**, or in the presence of extracellular matrix: Matrigel® **(B)**, collagen type I hydrogel **(C)**, collagen type I and laminin hydrogel **(D)**, and Vitrogel® **(E)**. Images were acquired using a phase-contrast microscope (Zeiss).
