# Supplementary material for: From inserts to 3D spheroids: MAC-T and BME-UV1 co-culture models for in vitro reconstruction of the bovine mammary epithelial architecture
Source: Vet Res. 2026 Jul 3;57:119. doi: 10.1186/s13567-026-01763-5 (PMC13332615; doi:10.1186/s13567-026-01763-5)
Supplement: Supplementary file 6 — Additional file 6. Phase-contrast imaging of BME-UV1 mammospheres after 7 days in different 3D culture conditions. BME-UV1 were cultured for 5 days in proliferation medium, followed by 2 days in differentiation medium,after which MAC-T cells were added and co-cultured in differentiation medium consisting of a 1:1 (v/v) mixture of therespective differentiation media. Cells were grown in different conditions: matrix-free ultra-low attachment plastic (A),or in the presence of extracellular matrix: Matrigel® (B), collagen type I hydrogel (C), and collagen type I and lamininhydrogel (D). Images were acquired using a phase-contrast microscope (Zeiss). [file 13567_2026_1763_MOESM6_ESM.docx]

### Additional file 6. Phase-contrast imaging of BME-UV1 mammospheres after 7 days in different 3D culture conditions


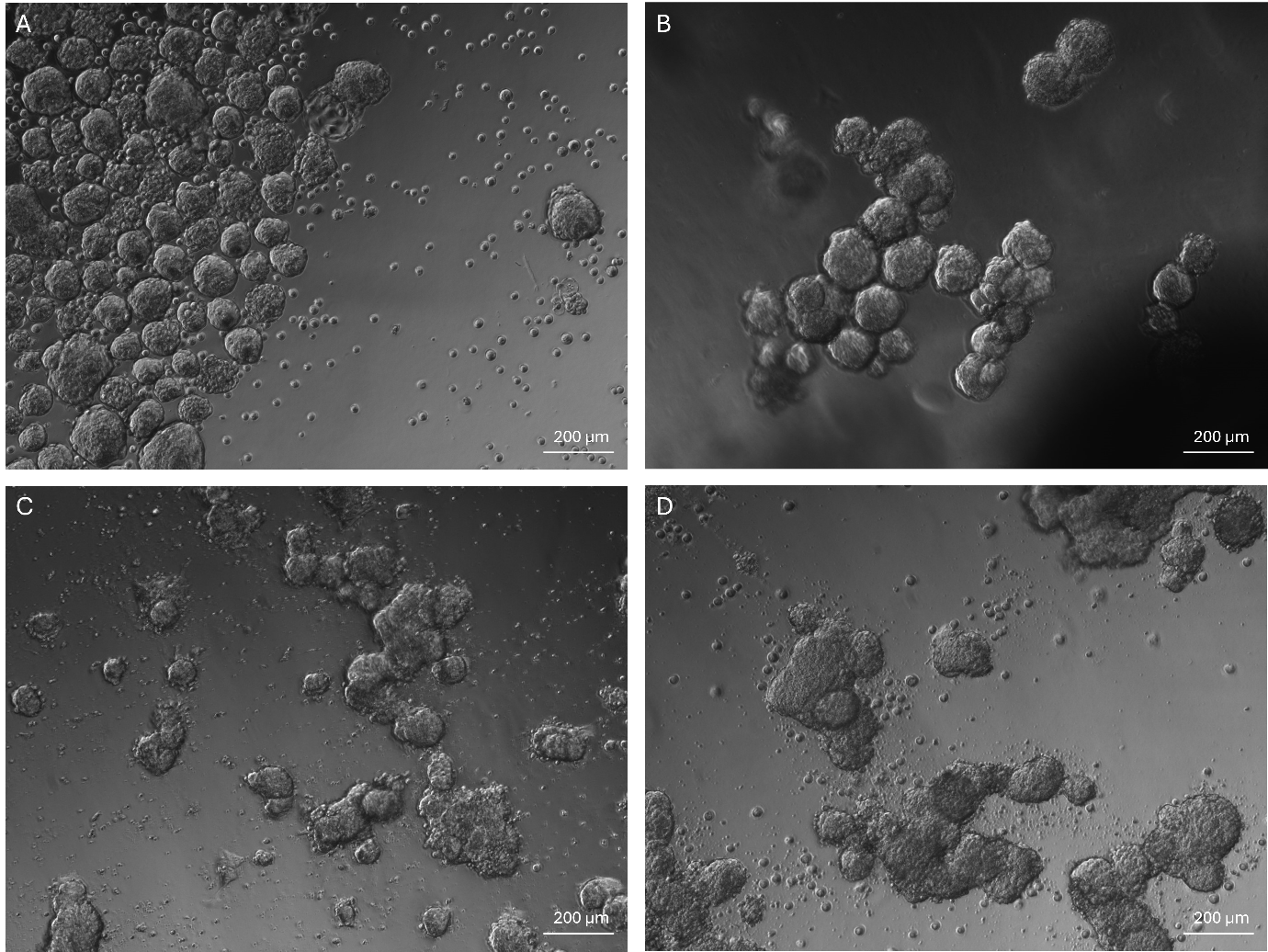


BME-UV1 were cultured for 5 days in proliferation medium, followed by 2 days in differentiation medium, after which MAC-T cells were added and co-cultured in differentiation medium consisting of a 1:1 (v/v) mixture of the respective differentiation media. Cells were grown in different conditions: matrix-free ultra-low attachment plastic **(A)**, or in the presence of extracellular matrix: Matrigel® **(B)**, collagen type I hydrogel **(C)**, and collagen type I and laminin hydrogel **(D)**. Images were acquired using a phase-contrast microscope (Zeiss)*.*
