# Supplementary material for: From inserts to 3D spheroids: MAC-T and BME-UV1 co-culture models for in vitro reconstruction of the bovine mammary epithelial architecture
Source: Vet Res. 2026 Jul 3;57:119. doi: 10.1186/s13567-026-01763-5 (PMC13332615; doi:10.1186/s13567-026-01763-5)
Supplement: Supplementary file 7 — Additional file 7. Phase-contrast imaging and mean size quantification of mammospheres from co-culturedBME-UV1 and MAC-T cells after 11 days in different 3D culture conditions. Cells were grown under different conditions: collagen type I hydrogel (A), collagen type I supplemented with laminin hydrogel (B), matrix-free ultra-lowattachment plastic (C), and Matrigel® (D). Continuous cell layers within the matrix are indicated with green arrows.Clusters of cells surrounding the BME-UV1 mammospheres are indicated with red arrows. Images were acquiredusing a phase-contrast microscope (Zeiss). Mammosphere diameter was quantified (E). Data are presented as mean± standard deviation; different letters (a-c) indicate statistically significant differences between culture conditions (p <0.05). [file 13567_2026_1763_MOESM7_ESM.docx]

### Additional file 7. Phase-contrast imaging and mean size quantification of mammospheres from co-cultured BME-UV1 and MAC-T cells after 11 days in different 3D culture conditions


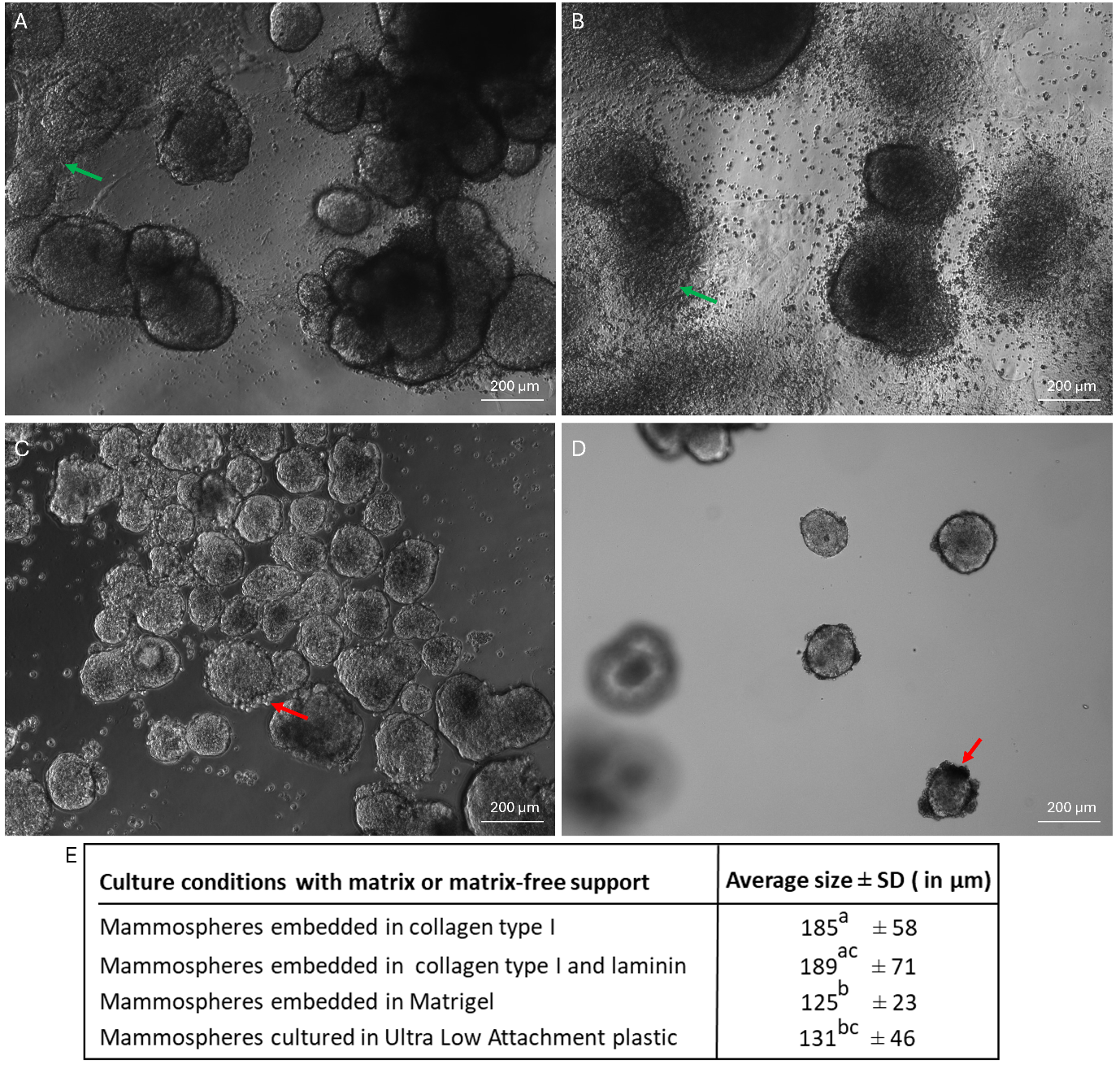


Cells were grown under different conditions: collagen type I hydrogel **(A)**, collagen type I supplemented with laminin hydrogel **(B)**, matrix-free ultra-low attachment plastic **(C)**, and Matrigel® **(D)**. Continuous cell layers within the matrix are indicated with green arrows. Clusters of cells surrounding the BME-UV1 mammospheres are indicated with red arrows. Images were acquired using a phase-contrast microscope (Zeiss). Mammosphere diameter was quantified **(E)**. Data are presented as mean ± standard deviation; different letters (a-c) indicate statistically significant differences between culture conditions (p < 0.05).
