# Supplementary material for: From inserts to 3D spheroids: MAC-T and BME-UV1 co-culture models for in vitro reconstruction of the bovine mammary epithelial architecture
Source: Vet Res. 2026 Jul 3;57:119. doi: 10.1186/s13567-026-01763-5 (PMC13332615; doi:10.1186/s13567-026-01763-5)
Supplement: Supplementary file 8 — Additional file 8. Kinetic phase-contrast imaging from seeding to day 11 of a single mammosphere from cocultured BME-UV1 and MAC-T cells. BME-UV1 mammosphere formation was monitored at 0, 8 and, 16 h (A-C),and at days 3 (D) and 4 (E) when cultured in proliferation medium. MAC-T cells were added to the BME-UV1mammosphere on day 7 (F) and co-cultured in differentiation medium consisting of a 1:1 (v/v) mixture of therespective differentiation media. The co-cultured mammosphere was subsequently visualized 8 h (G) and 16 h (H)after MAC-T addition, and further monitored on days 8 (I), 9 (J), 10 (K), and 11 (L). Red arrows indicate MAC-T cells.Images were acquired using a BioTek Cytation 5 Cell Imaging Multimode Reader (Agilent Technologies). [file 13567_2026_1763_MOESM8_ESM.docx]

### Additional file 8. Kinetic phase-contrast imaging from seeding to day 11 of a single mammosphere from co-cultured BME-UV1 and MAC-T cells


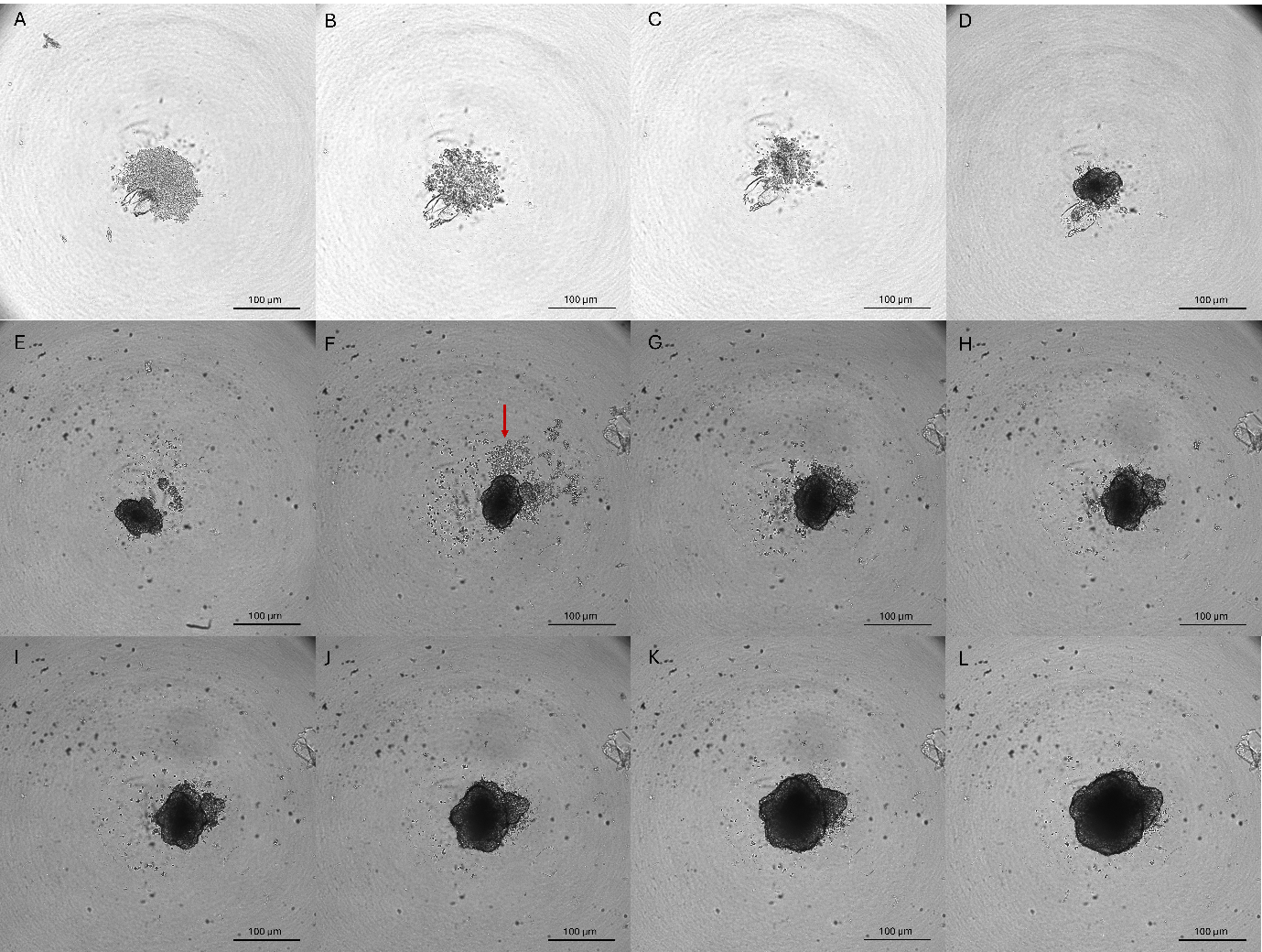


BME-UV1 mammosphere formation was monitored at 0, 8 and, 16 h **(A-C)**, and at days 3 **(D)** and 4 **(E)** when cultured in proliferation medium. MAC-T cells were added to the BME-UV1 mammosphere on day 7 **(F)** and co-cultured in differentiation medium consisting of a 1:1 (v/v) mixture of the respective differentiation media. The co-cultured mammosphere was subsequently visualized 8 h **(G)** and 16 h **(H)** after MAC-T addition, and further monitored on days 8 **(I)**, 9 **(J)**, 10 **(K)**, and 11 **(L)**. Red arrows indicate MAC-T cells. Images were acquired using a BioTek Cytation 5 Cell Imaging Multimode Reader (Agilent Technologies).
