# Supplementary material for: From inserts to 3D spheroids: MAC-T and BME-UV1 co-culture models for in vitro reconstruction of the bovine mammary epithelial architecture
Source: Vet Res. 2026 Jul 3;57:119. doi: 10.1186/s13567-026-01763-5 (PMC13332615; doi:10.1186/s13567-026-01763-5)
Supplement: Supplementary file 9 — Additional file 9. Zonula Occludens 1 (ZO-1) labeling of mammospheres from co-cultured BME-UV1 and MAC-T cells after 11 days of culture. Mammosphere from co-cultured BME-UV1 and MAC-T cells in ultra-low attachmentplates (A) and Matrigel® (B) were labeled with anti-ZO-1 (red) and nuclei with Hoechst 33342 (blue). Z-stack imagingand three-dimensional reconstructions were performed to provide an overview of ZO-1 distribution throughout theentire mammosphere. Images were acquired by confocal laser scanning microscope (CLSM, LSM880, Zeiss) usingZen software (Zeiss). [file 13567_2026_1763_MOESM9_ESM.docx]

### Additional file 9: Zonula Occludens 1 (ZO-1) labeling of mammospheres from co-cultured BME-UV1 and MAC-T cells after 11 days of culture


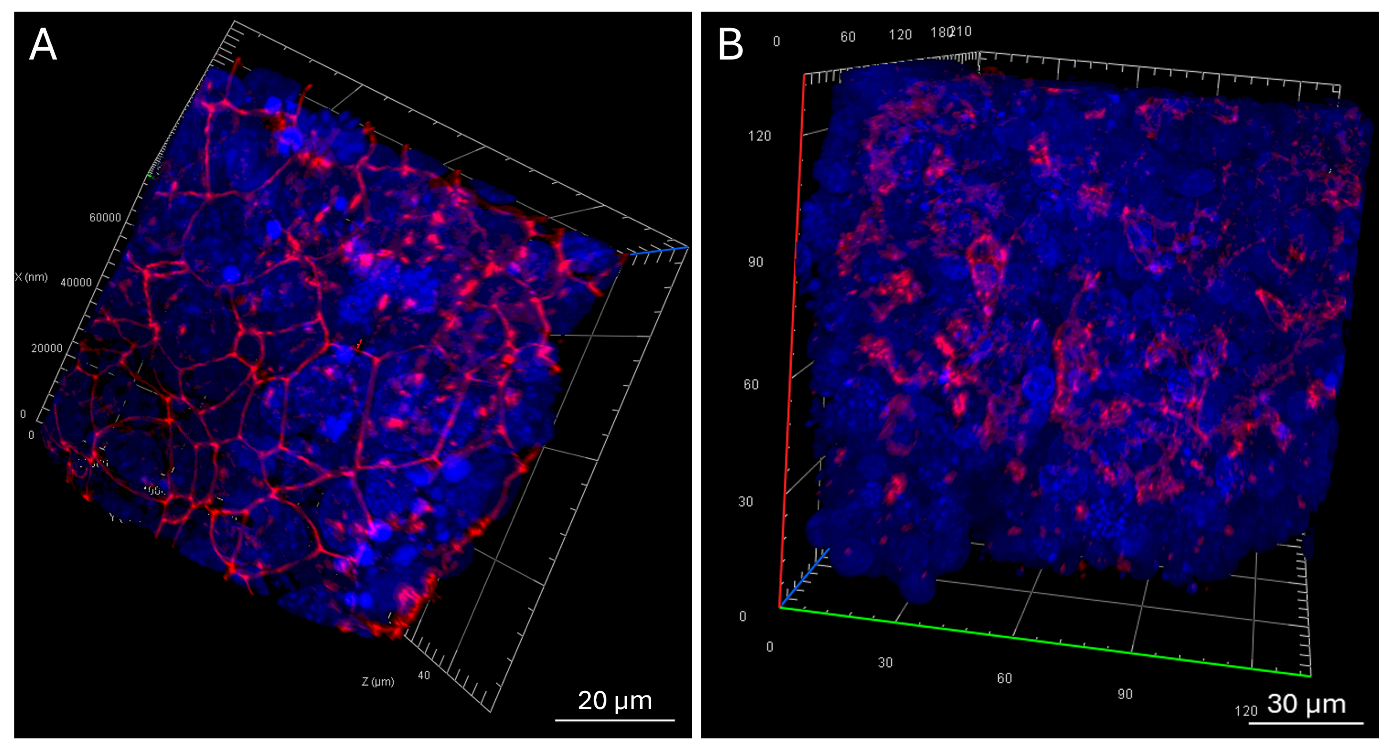


Mammosphere from co-cultured BME-UV1 and MAC-T cells in ultra-low attachment plates **(A)** and Matrigel® **(B)** were labeled with anti-ZO-1 (red) and nuclei with Hoechst 33342 (blue). Z-stack imaging and three-dimensional reconstructions were performed to provide an overview of ZO-1 distribution throughout the entire mammosphere. Images were acquired by confocal laser scanning microscope (CLSM, LSM880, Zeiss) using Zen software (Zeiss).
