# Supplementary material for: From inserts to 3D spheroids: MAC-T and BME-UV1 co-culture models for in vitro reconstruction of the bovine mammary epithelial architecture
Source: Vet Res. 2026 Jul 3;57:119. doi: 10.1186/s13567-026-01763-5 (PMC13332615; doi:10.1186/s13567-026-01763-5)
Supplement: Supplementary file 10 — Additional file 10. Viability assessment of single mammosphere from co-cultured BME-UV1 and MAC-T cellsin Ultra-Low-Attachment (ULA) conditions after 11 days of culture.Mammosphere from co-cultured BME-UV1and MAC-T cells in ultra-low attachment plates at 11 days of culture were assessed for cell viability using twocomplementary approaches: Live imaging of a non-fixed mammosphere stained with propidium iodide (PI; red) andHoechst 33342 (blue) (A); Live/Dead viability assay performed on a second mammosphere using the Viability™488/520 Fixable Dye (green) in combination with Hoechst 33342 (blue) (B). Images were acquired by fluorescencemicroscopy using a THUNDER epifluorescence microscope (Leica). [file 13567_2026_1763_MOESM10_ESM.docx]

Additional file 10: Viability assessment of single mammosphere from co-cultured BME-UV1 and MAC-T cells in Ultra-Low-Attachment (ULA) conditions after 11 days of culture


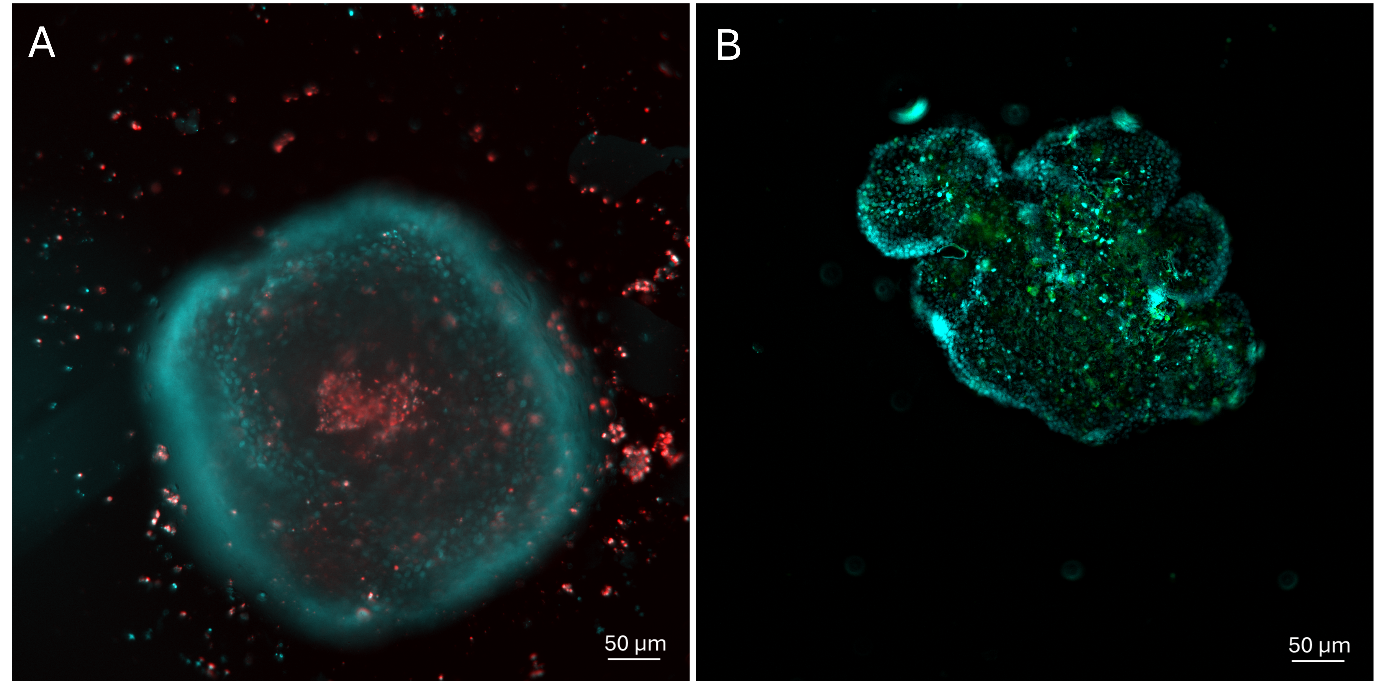


Mammosphere from co-cultured BME-UV1 and MAC-T cells in ultra-low attachment plates at 11 days of culture were assessed for cell viability using two complementary approaches: Live imaging of a non-fixed mammosphere stained with propidium iodide (PI; red) and Hoechst 33342 (blue) **(A)**; Live/Dead viability assay performed on a second mammosphere using the Viability™ 488/520 Fixable Dye (green) in combination with Hoechst 33342 (blue) **(B)**. Images were acquired by fluorescence microscopy using a THUNDER epifluorescence microscope (Leica).
